# Supplementary material for: Centrosome amplification mediates small extracellular vesicle secretion via lysosome disruption
Source: Curr Biol. 2021 Apr 12;31(7):1403–1416.e7. doi: 10.1016/j.cub.2021.01.028 (PMC8047808; doi:10.1016/j.cub.2021.01.028)
Supplement: Document S1. Figures S1–S5 and Table S1 [file mmc1.pdf]

Current Biology, Volume *31*

Supplemental Information

Centrosome amplification mediates small extracellular vesicle secretion via lysosome disruption

Sophie D. Adams, Judit Csere, Gisela D'angelo, Edward P. Carter, Maryse Romao, Teresa Arnandis, Martin Dodel, Hemant M. Kocher, Richard Grose, Graça Raposo, Faraz Mardakheh, and Susana A. Godinho

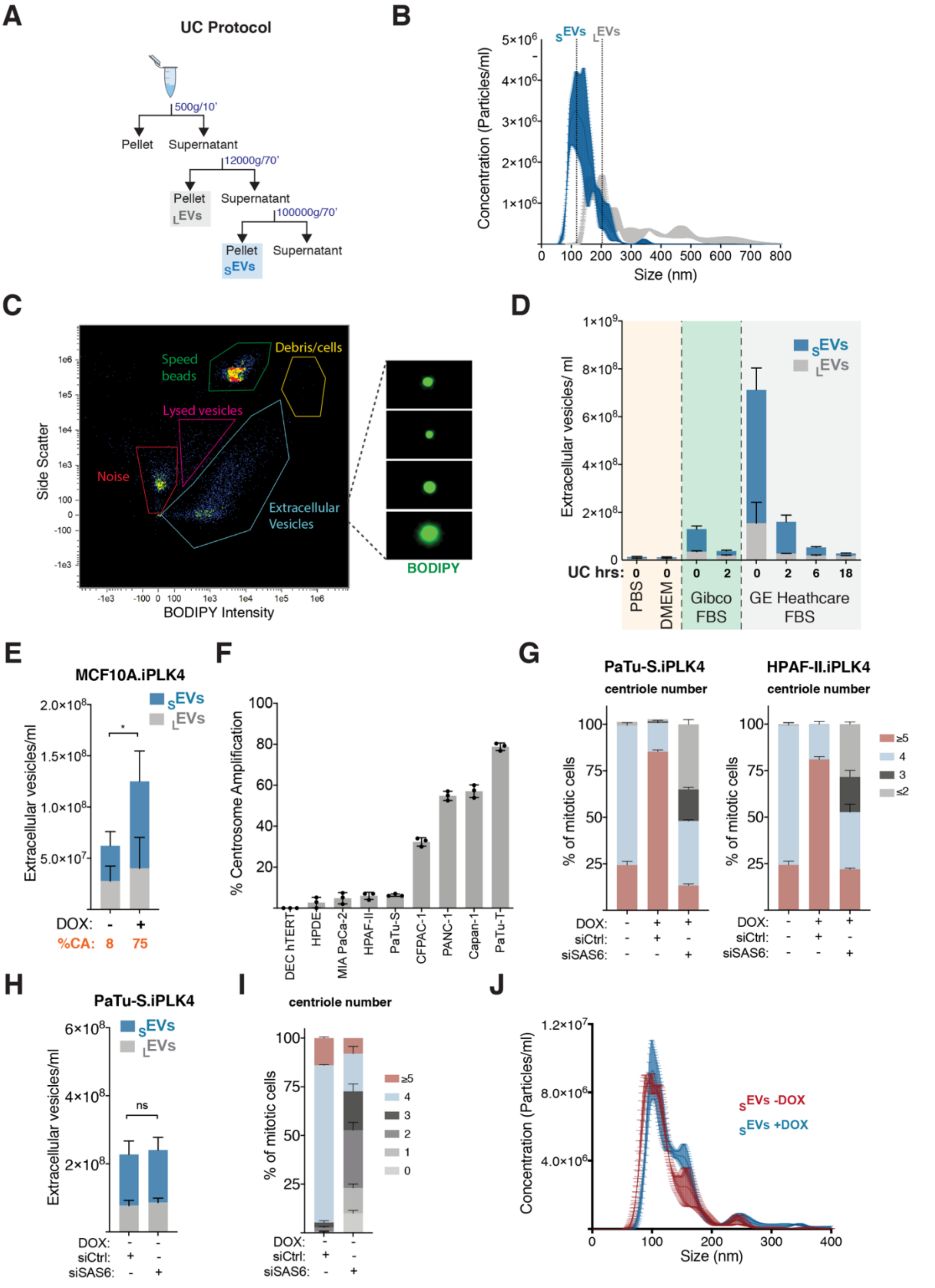

Figure S1. EV isolation and characterization in PDAC cell lines. Related to Figure 1. (A) Experimental flowchart. (B) Quantification of $_{S}$EVs and $_{L}$EVs concentration and size using the nanoparticle tracking device NanoSight to assess the reliability of the UC protocol to separate EVs by size. (C) Example scatterplot from ImageStream displaying side scatter plotted against BODIPY maleimide intensity. Representative gating regions are shown. Gating region for contaminating cells and cell debris (yellow), speed beads (green) used to internally calibrate the ImageStream and for lysed vesicles (purple) are shown. Representative images or particles taken from the ImageStream image gallery that are present in the EV gating region show spherical BOPIDY-labelled vesicles. (D) Quantification of $_{S}$EVs and $_{L}$EVs in the reagents used to culture cells and

prepare EVs for analyses and after UC to determine removal of any contaminant EV. (E) Quantification of secreted $_{S}$EVs and $_{L}$EVs from MCF10A.iPLK4 cell line upon induction of centrosome amplification (+DOX). Average of the percentage of centrosome amplification (CA) per cell line is highlighted in orange. (F) Quantification of the percentage of centrosome amplification in a panel of PDAC cell lines. n=300 mitotic cells for each cell line. (G) Quantification of the number of centrioles in the PaTu-S.iPLK4 (left) and HPAF-II.iPLK4 (right) cell lines upon induction of centrosome amplification (+DOX) and Sas-6 depletion by siRNA. n=300 mitotic cells for each condition. (H) Quantification of secreted $_{S}$EVs and $_{L}$EVs from PaTu-S.iPLK4 control cells (-DOX) upon depletion of Sas-6. 4= normal and ≥5 centrioles per mitotic cell = centrosome amplification. (I) Quantification of the number of centrioles in PaTu-S.iPLK4 cells upon depletion of Sas-6. n=300 mitotic cells for each condition. (J) Quantification of the size of $_{S}$EVs secreted by PaTu-S.iPLK4 cells with (+DOX) and without (-DOX) extra centrosomes using the NanoSight. For all graphics error bars represent mean +/- SD from three independent experiments. *$p < 0.05$, ****$p < 0.0001$, *n*.s. = not significant ($p > 0.05$). The following statistic were applied: for graph in E two-way ANOVA with Tukey's post hoc test was applied and for graphs in H unpaired *t* test was applied.

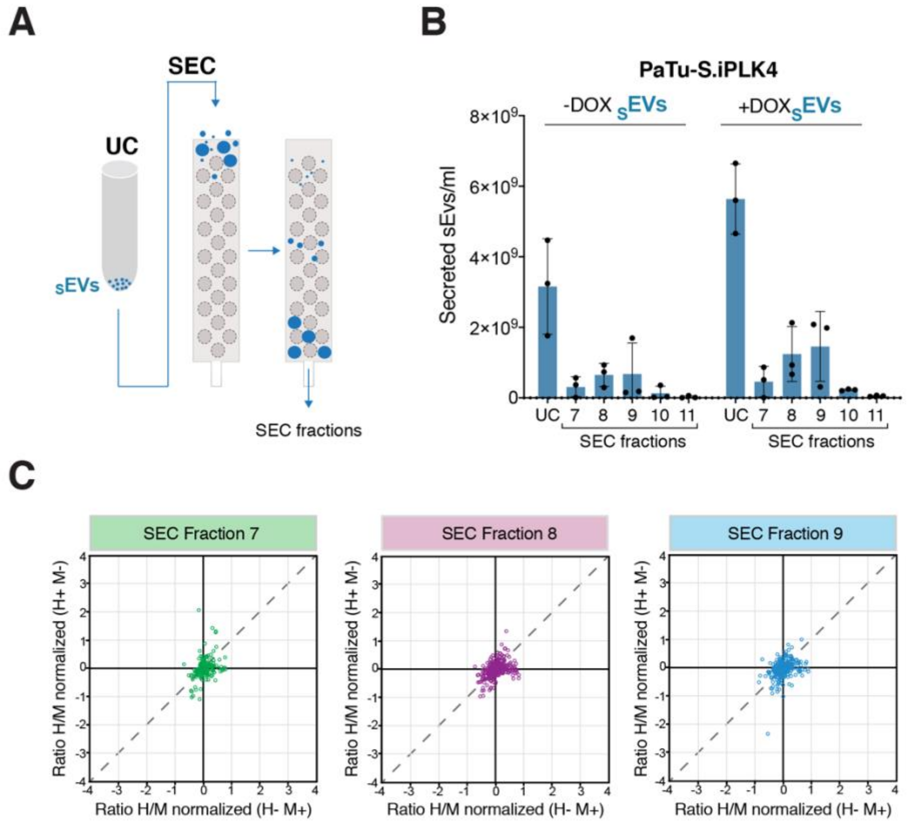

D

SEC Fraction 7			
**Gene name**	**-DOX sEVs**	**+DOX sEVs**	**Peptide number**
KIF5B	✗	✓	42
B2M	✗	✓	3
CYBRD1	✗	✓	1
ERLIN2	✗	✓	13
SLC25A3	✗	✓	14
OCLN	✗	✓	5
ANXA3	✗	✓	19
MAPK1IP1L	✓	✗	1

E

SEC Fraction 8			
**Gene name**	**-DOX sEVs**	**+DOX sEVs**	**Peptide number**
CD81	✓	✗	3
PGM3	✗	✓	8
CAD	✗	✓	48
MT-CO2	✗	✓	3
NIBAN	✗	✓	9
CCDC124	✗	✓	8

Figure S2. SILAC proteomic analyses of secreted $_S$EVs. Related to Figure 2. (A) Experimental flowchart. (B) Quantification of number $_S$EVs in UC and SEC fractions collected from PaTu-S.iPLK4 cells with (+DOX) and without (-DOX) extra centrosomes. (C) Correlation graphs plotting Log_2 fold change of the ratio of heavy (H) and medium (M) labelled proteins of the forward and reverse experiments for the SEC fractions 7, 8 and 9. Dashed diagonal line illustrates where identical M and H would lie, demonstrating the similarity between H and M labelled $_S$EVs. (D) Table with the proteins that were lost/gain in SEC fraction 7 of $_S$EVs secreted by cells with extra centrosomes (+DOX). (E) Table with the proteins that were lost/gain in SEC fraction 8 of $_S$EVs secreted by cells with extra centrosomes (+DOX). See also Table S4.

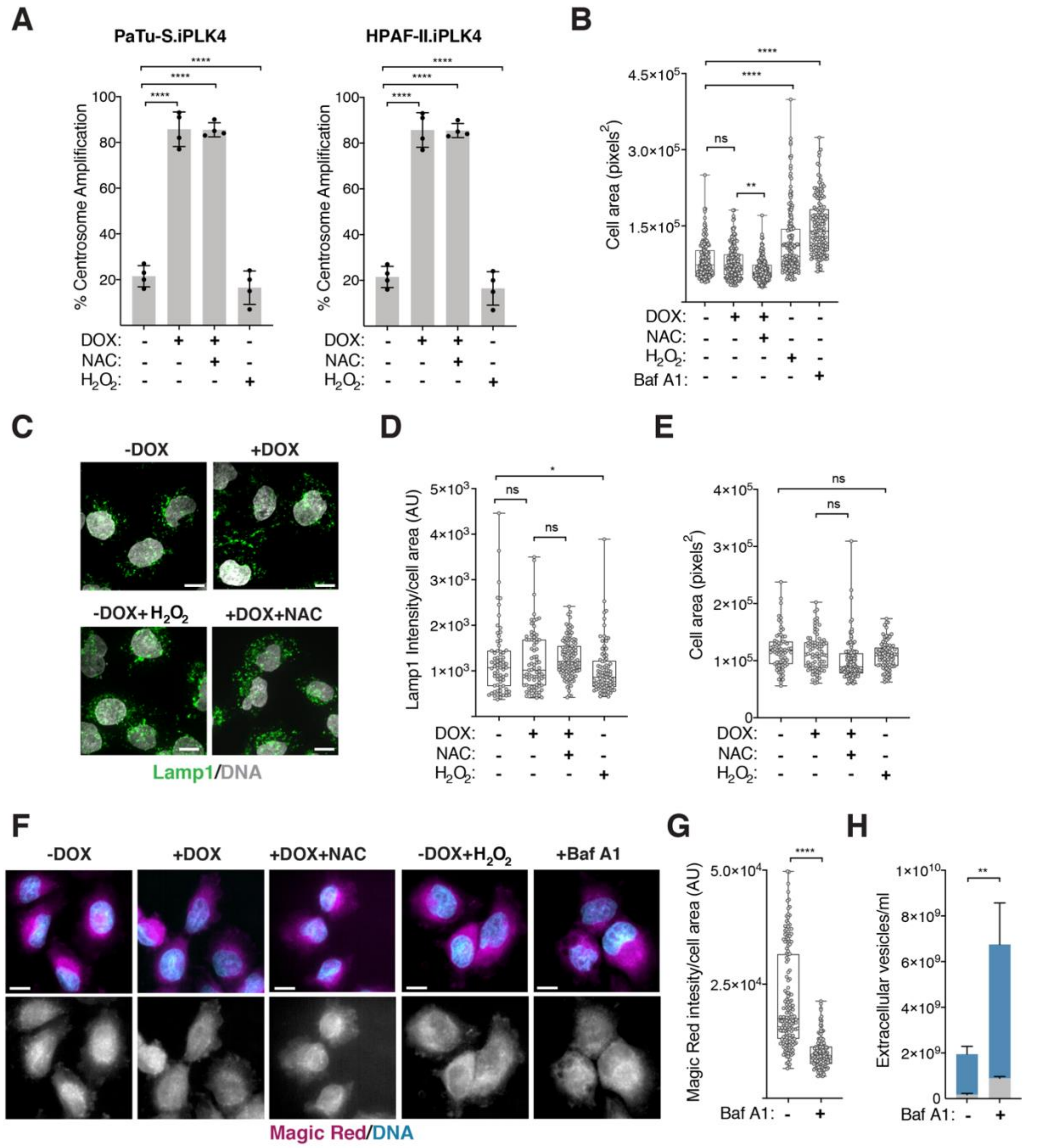

Figure S3. Characterization of lysosome function in cells with amplified centrosomes. Related to Figure 3. (A) Quantification of centrosome amplification in the PaTu-S.iPLK4 (left) and HPAF-II.iPLK4 (right) cell lines upon induction of centrosome amplification (+DOX). 5 mM of NAC, 100 μM H_2O_2 and 20 nM Baf A1 was used. n=300 mitotic cell lines for each condition. (B) Quantification of cell area (pixels^2). 5 mM of NAC, 100 μM H_2O_2 and 20 nM Baf A1 was used. $n_{(-DOX)}$=158, $n_{(+DOX)}$=189, $n_{(+DOX+NAC)}$=221, $n_{(-DOX+H2O2)}$=175 and $n_{(+BafA1)}$=144. (C) Representative confocal images of PaTu-S.iPLK4 cells stained for total lysosomes (Lamp1, green) and DNA (grey). Scale bar, 10 μm. (D) Quantification of Lamp1 fluorescence intensity in PaTu-S.iPLK4 cells normalized for cell area. 5 mM of NAC, 100 μM H_2O_2 was used. AU, arbitrary units. $n_{(-DOX)}$=71, $n_{(+DOX)}$=80, $n_{(+DOX+NAC)}$=112 and $n_{(-DOX+H2O2)}$=87. (E) Quantification of cell area (pixels^2). 5 mM of NAC, 100 μM H_2O_2 was used. $n_{(-DOX)}$=71, $n_{(+DOX)}$=80, $n_{(+DOX+NAC)}$=112 and $n_{(-DOX+H2O2)}$=87. (F) Representative confocal images of PaTu-S.iPLK4 cells stained for functional lysosomes (Magic red, magenta) and DNA (cyan). SUM projection images used for fluorescence intensity quantification. Baf A1 was used at 20 nM. Scale bar, 10 μm. (G) Quantification of intracellular Magic red fluorescence intensity normalized for cell area in PaTu-S.iPLK4 cells. AU, arbitrary units. $n_{(control)}$=158 and $n_{(+BafA1)}$=144. Note that data plotted for control cells is the same as in Figure 3D. (H) Quantification of $_S$EVs and $_L$EVs secretion in control and Baf A1 treated PaTu-

S.iPLK4 cells. For all graphics error bars represent mean +/- SD from three independent experiments. *$p < 0.05$, **$p < 0.01$, ****$p < 0.0001$, n.s. = not significant ($p > 0.05$). The following statistic were applied: for graphs in A, one-way ANOVA with Tukey's post hoc test was applied, for graphs in B, D, E and G data one-way ANOVA with a Kruskal-Wallis post hoc test was applied and for data in H two-way ANOVA with Tukey's post hoc test was applied.

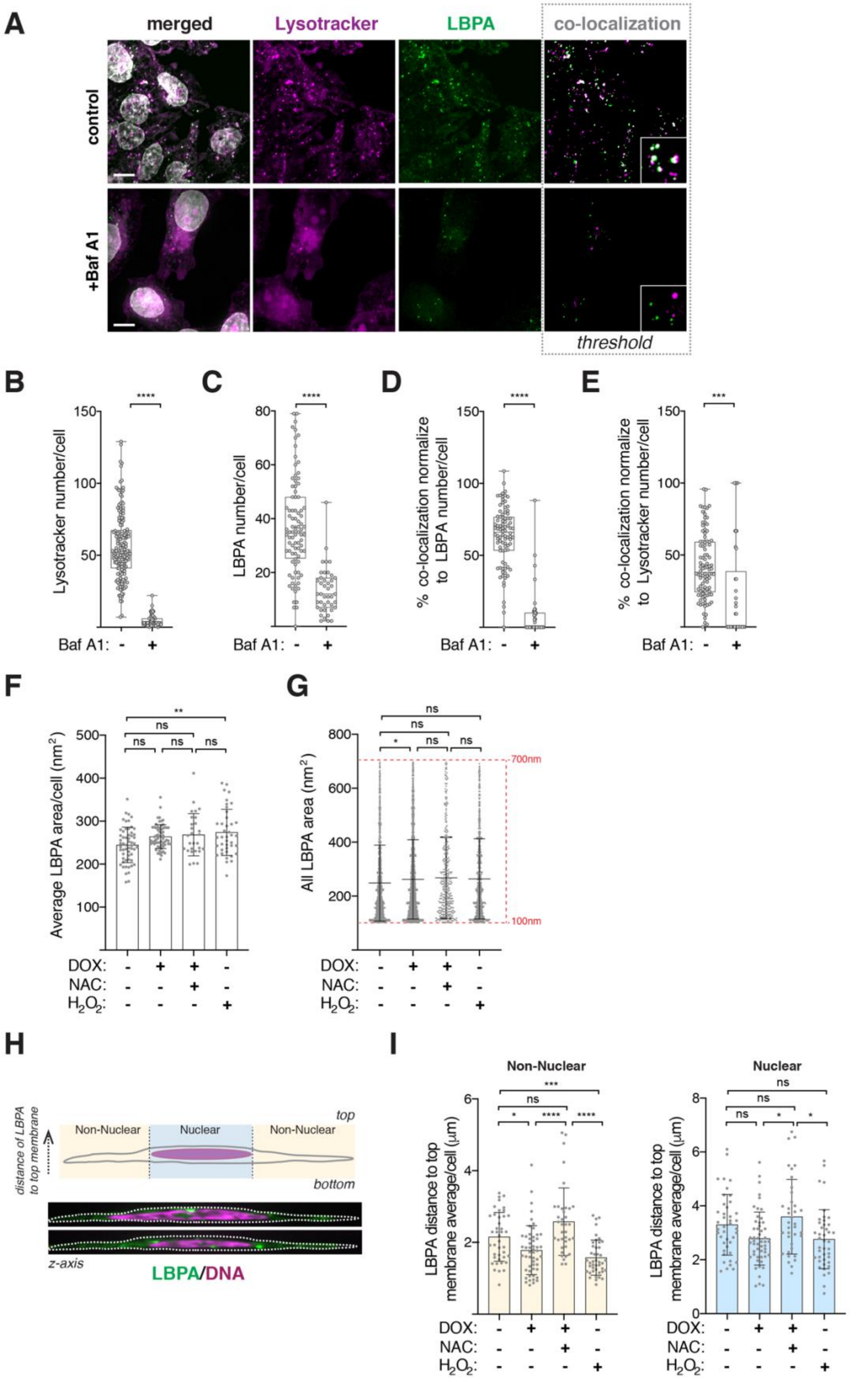

Figure S4. Characterization of Bafilomycin A1 treatment and analyses of MVB dispersion and localization. Related to Figure 4. (A) Representative confocal images of cells stained for acidic lysosomes (Lysotracker, magenta), late endosomes/MVBs (anti-LBPA, green) and DNA (grey). Insets show higher magnification of lysotracker and LBPA-labelled vesicles. Scale bar, 10 μm. (B) Quantification of the number of lysotracker-labelled lysosomes per cell. 20 nM of Baf A1 was used. $n_{(control)}$=166 and $n_{(+BafA1)}$=67. Note

that data plotted for control cells is the same as in Figure 4B. (C) Quantification of LBPA-labelled late endosomes/MVBs per cell. 20 nM of Baf A1 was used. $n_{(control)}$=88 and $n_{(+BafA1)}$=42. Note that data plotted for control cells is the same as in Figure 4C. (D) Quantification of the percentage of lysotracker and LBPA-labelled intracellular vesicles co-localization. 20 nM of Baf A1 was used. $n_{(control)}$=86 and $n_{(+BafA1)}$=42. (E) Quantification of the percentage of lysotracker and LBPA-labelled intracellular vesicles co-localization normalized to lysotracker number. 20 nM of Baf A1 was used. $n_{(control)}$=86 and $n_{(+BafA1)}$=42. (F) Quantification of the average size of LBPA vesicles per cell. $n_{(-DOX)}$=57, $n_{(+DOX)}$=63, $n_{(+DOX+NAC)}$=28 and $n_{(-DOX+H2O2)}$=40. (G) Quantification of the size of all LBPA vesicles. (H) Representative image depicting method for quantifying LPBA-membrane distance. Cells were stained for LBPA (green) and DNA (magenta). Distance is calculated to the top membrane, opposite to coverslip. (I) Left: Quantification of the average LBPA-membrane distance per cell in the non-nuclear region. Right: Quantification of the average LBPA-membrane distance per cell in the nuclear. $n_{(-DOX)}$=41, $n_{(+DOX)}$=53, $n_{(+DOX+NAC)}$=38 and $n_{(-DOX+H2O2)}$=44. Note that data plotted for control cells is the same as in Figure 4D. For all graphics error bars represent mean +/- SD from three independent experiments. *$p < 0.05$, **$p < 0.01$, ***$p < 0.001$****$p < 0.0001$,* n.s. = not significant ($p > 0.05$). Graphs in B-E were analyzed with unpaired *t* test, graphs in G, and I were analyzed with one-way ANOVA with a Kruskal-Wallis post hoc test and for graph in F one-way ANOVA with Tukey's post hoc test was used.

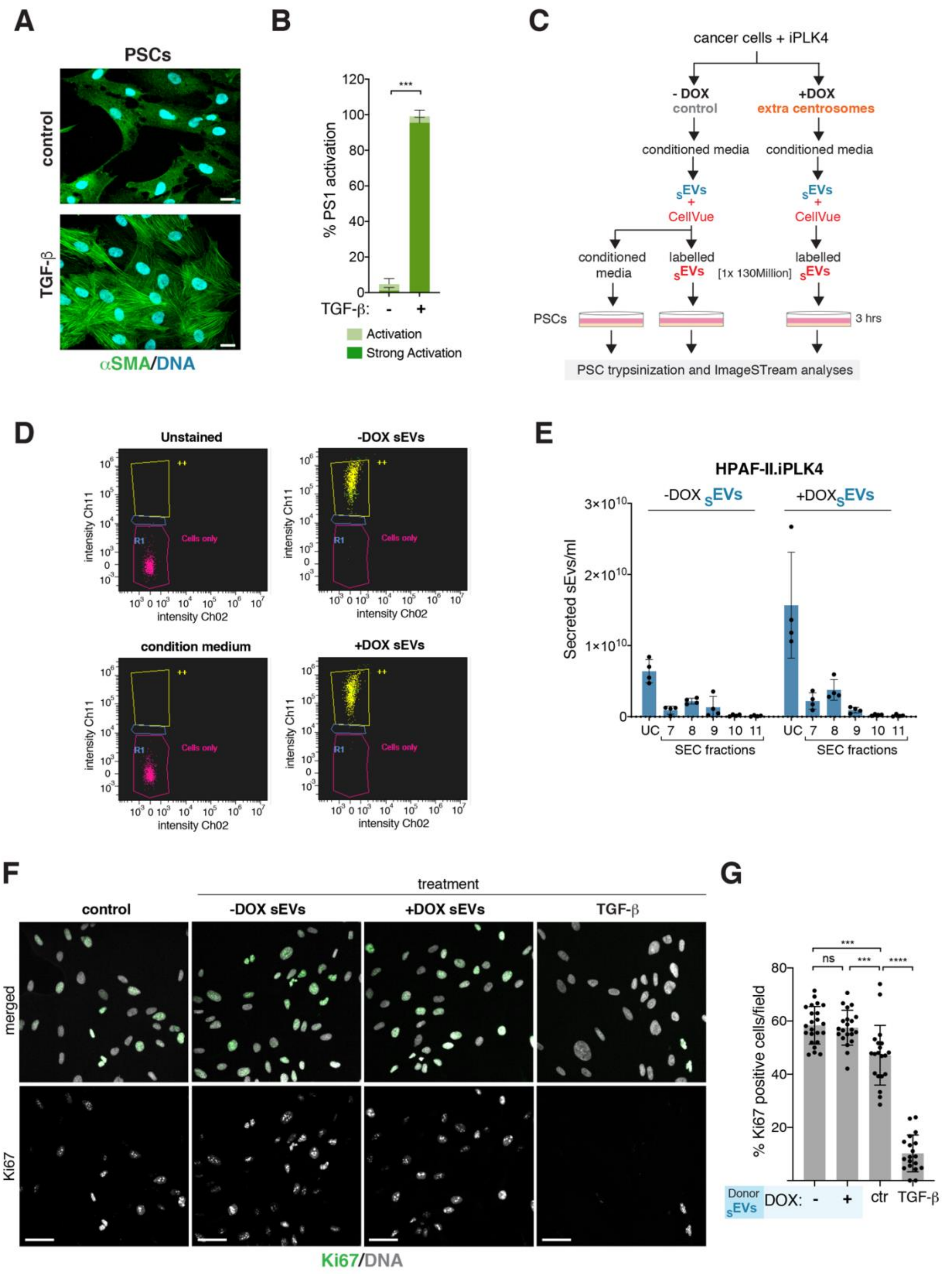

Figure S5. Characterization of PSCs activation. Related to Figure 5. (A) Representative confocal images of PSCs stained for α-SMA (green) and DNA (cyan). Scale bar, 20 μm. (B) Quantification of the percentage of PSCs activation upon treatment with TGF-β, used as positive control. 5 ng/ml of TGF-β was used. PSCs $n_{(control)}$=475, $n_{(+TGF-\beta)}$=414. (C) Schematic representation of the $_S$EVs uptake experiment. (D) Examples of scatterplots from ImageStream displaying unlabeled cells side scatter plotted against CellVue intensity. Representative gating regions are shown. Gating region for CellVue positive (yellow), unlabeled cells (magenta) were used to determine the percentage of cells that internalized CellVue-labelled $_S$EVs. (E) Quantification of number $_S$EVs in UC and SEC fractions collected from HPAF-II.iPLK4 cells with (+DOX) and

without (-DOX) extra centrosomes. (F) Representative confocal images of PS1 cells stained for the proliferation marker Ki67 (green) and DNA (grey). Scale bar, 50 μm. Quantification of the percentage of Ki67 positive cells per total cells in different image fields. $n_{(control)}$=842, $n_{(-DOX\ sEVs)}$=1049, $n_{(+DOX\ sEVs)}$=996 and $n_{(+TGF-\beta)}$=854. For all graphics error bars represent mean +/- SD from three independent experiments. ****p < 0.001*. For graph in B aired *t* test was used for statistical analyses and for graph in G two-way ANOVA with Tukey's post hoc test was used.

Cell Line	Platting density in 15 cm dishes	Final cell count at end point
DEC-hTERT	3.2 x10^6	~6.4 x10^6
HPDE	3.6 x10^6	~6.4 x10^6
MIA-PaCa-2	1.32 x106	~6.4 x10^6
HPAF-II	1.32 x10^6	~6.4 x10^6
PaTu-S	7.5 x10^5	~6.4 x10^6
CFPAC-1	1.44 x10^6	~6.4 x10^6
Panc-1	2.16 x10^6	~6.4 x10^6
Capan-1	2.52 x10^6	~6.4 x10^6
PaTu-T	1.2 x106	~6.4 x10^6
PaTu-S.iPLK4 -DOX	7.5 x10^5	~6.4 x10^6
PaTu-S.iPLK4 + 48 hrs DOX	8 x10^5	~6.4 x10^6
HPAF-II.iPLK4 -DOX	1.32 x10^6	~6.4 x10^6
HPAF-II.iPLK4 + 48 hrs DOX	1.35 x10^6	~6.4 x10^6

Table S1. Cell platting conditions. Related to Figure 1. Platting density in 15 cm dishes utilized for the different cell lines and conditions to ensure a similar final cell number at end point, when EVs are collected for analyses. Note that for all experiments, final cell numbers were always assessed.
