# Supplementary material for: Centrosome amplification mediates small extracellular vesicle secretion via lysosome disruption
Source: Curr Biol. 2021 Apr 12;31(7):1403–1416.e7. doi: 10.1016/j.cub.2021.01.028 (PMC8047808; doi:10.1016/j.cub.2021.01.028)
Supplement: Document S2. Article plus supplemental information [file mmc6.pdf]

Article

Current Biology

Centrosome amplification mediates small extracellular vesicle secretion via lysosome disruption

Highlights

- Centrosome amplification induces $_S$EV secretion
- Proteomic analysis suggests that secreted $_S$EVs are of endocytic origin
- Lysosomal dysfunction leads to $_S$EV secretion in cells with extra centrosomes
- $_S$EVs secreted by PDAC with extra centrosome activate pancreatic stellate cells

Authors

Sophie D. Adams, Judit Csere, Gisela D'angelo, ..., Graça Raposo, Faraz Mardakheh, Susana A. Godinho

Correspondence

s.godinho@qmul.ac.uk

In Brief

Adams et al. demonstrate that lysosome dysfunction downstream of centrosome amplification increases the secretion of small extracellular vesicles ($_S$EVs). These $_S$EVs are functionally distinct and activate fibroblast-like cells, suggesting that cancer cells with amplified centrosomes could change the tumor microenvironment.

Adams et al., 2021, Current Biology *31*, 1403–1416
April 12, 2021 © 2021 The Authors. Published by Elsevier Inc.
https://doi.org/10.1016/j.cub.2021.01.028

CellPress

Current Biology

CellPress
OPEN ACCESS

Article

Centrosome amplification mediates small extracellular vesicle secretion via lysosome disruption

Sophie D. Adams,[1] Judit Csere,[1] Gisela D'angelo,[2] Edward P. Carter,[3] Maryse Romao,[2] Teresa Arnandis,[1,4] Martin Dodel,[1] Hemant M. Kocher,[3] Richard Grose,[3] Graça Raposo,[2] Faraz Mardakheh,[1] and Susana A. Godinho[1,5,6,*]

[1]Centre for Cancer Cell and Molecular Biology, Barts Cancer Institute, Queen Mary University of London, Charterhouse Square, London EC1M 6BQ, UK

[2]Structure and Membrane Compartments, Institute Curie, Paris Sciences & Lettres Research University, Centre for National de la Recherche Scientifique, UMR144, Paris, France

[3]Centre for Tumour Biology, Barts Cancer Institute, Queen Mary University of London, Charterhouse Square, London EC1M 6BQ, UK

[4]Present address: Department of Pathology, School of Medicine and Dentistry, Catholic University of Valencia, 46001 Valencia, Spain

[5]Twitter: @godinho_susana

[6]Lead contact

*Correspondence: s.godinho@qmul.ac.uk

https://doi.org/10.1016/j.cub.2021.01.028

SUMMARY

Bidirectional communication between cells and their surrounding environment is critical in both normal and pathological settings. Extracellular vesicles (EVs), which facilitate the horizontal transfer of molecules between cells, are recognized as an important constituent of cell-cell communication. In cancer, alterations in EV secretion contribute to the growth and metastasis of tumor cells. However, the mechanisms underlying these changes remain largely unknown. Here, we show that centrosome amplification is associated with and sufficient to promote small extracellular vesicle ($_S$EV) secretion in pancreatic cancer cells. This is a direct result of lysosomal dysfunction, caused by increased reactive oxygen species (ROS) downstream of extra centrosomes. We propose that defects in lysosome function could promote multivesicular body fusion with the plasma membrane, thereby enhancing $_S$EV secretion. Furthermore, we find that $_S$EVs secreted in response to amplified centrosomes are functionally distinct and activate pancreatic stellate cells (PSCs). These activated PSCs promote the invasion of pancreatic cancer cells in heterotypic 3D cultures. We propose that $_S$EVs secreted by cancer cells with amplified centrosomes influence the bidirectional communication between the tumor cells and the surrounding stroma to promote malignancy.

INTRODUCTION

A variety of human cancer types often exhibit defects in the structure and number of centrosomes, the main microtubule organizing centers in animal cells.[1,2] Work in fly and mouse models has shown that centrosome abnormalities, in particular centrosome amplification, are not mere byproducts of tumorigenesis but rather play direct roles in promoting and accelerating tumor progression.[3–6] Although the full extent by which centrosome abnormalities contribute to tumorigenesis is still unclear, centrosome amplification can directly promote aneuploidy and cell invasion, which play important roles in malignant progression.[7–9] Recently, we reported that centrosome amplification induces the secretion of several proteins with pro-invasive properties, e.g., interleukin-8, which induces invasive behavior in neighboring cells.[10] This altered secretion is partially due to a stress response that results from increased reactive oxygen species (ROS) downstream of centrosome amplification.[10] Thus, the presence of amplified centrosomes can also influence tumors in a non-cell-autonomous manner, via protein secretion, suggesting a broader and more complex role for these abnormalities in cancer.

Secretion of cytokines, growth factors, and extracellular vesicles (EVs) promotes bidirectional communication between cancer cells and the tumor microenvironment. This cross-talk impacts tumor initiation, progression, and patient prognosis.[11,12] EVs are membrane-bound vesicles containing proteins, lipids, DNA, and RNA species (microRNA, mRNA, and long non-coding RNAs) that can mediate the horizontal transfer of molecules between cells.[13] Their role in cell-cell communication is particularly interesting due to their suspected long-lasting effects and ability to influence distant tissues, e.g., during pre-metastatic niche formation.[14] Eukaryotic cells secrete two main types of EVs, microvesicles and exosomes, which differ in their size and biogenesis pathways. Microvesicles (large EVs [$_L$EVs]; ~100–1,000 nm diameter) are formed through outward budding or "shedding" of the plasma membrane. In comparison, exosomes (small EVs [$_S$EVs]; ~30–150 nm diameter) are generated intracellularly as intraluminal vesicles within multivesicular bodies, which are released upon the fusion of the multivesicular body with the plasma membrane.[13] Both types of EVs are secreted by cancer cells and have been shown to play key roles in tumor progression, potentially via changes in their composition.[15,16]

This is an open access article under the CC BY license (http://creativecommons.org/licenses/by/4.0/).

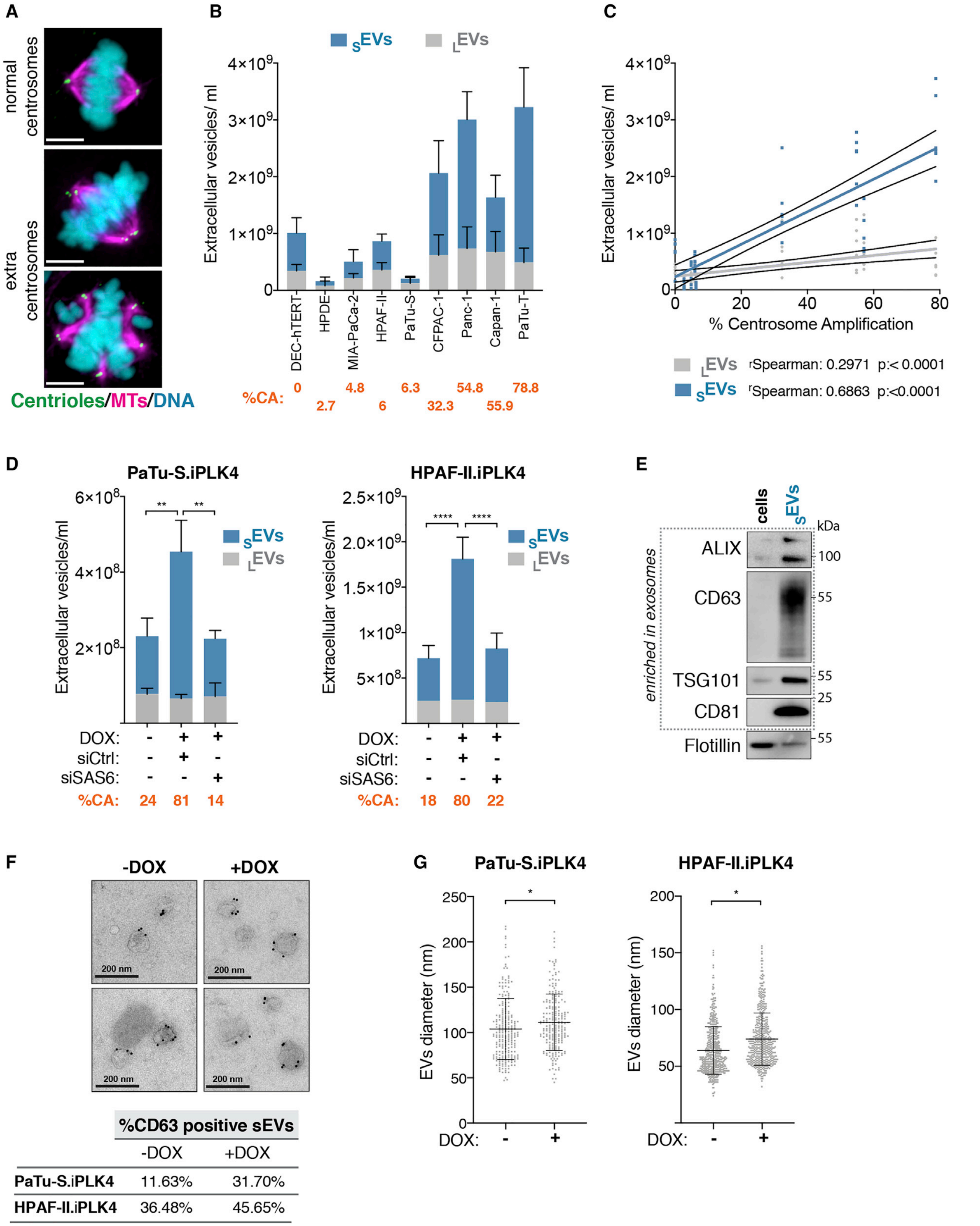

%CD63 positive sEVs	-DOX	+DOX
PaTu-S.iPLK4	11.63%	31.70%
HPAF-II.iPLK4	36.48%	45.65%

Figure 1. Centrosome amplification promotes the secretion of $_{S}$EVs in PADC cells

(A) Representative confocal images of mitotic cells with normal and amplified centrosomes. Cells were stained for α-tubulin (magenta), centrin2 (green), and DNA (cyan). Scale bar, 10 μm.

(legend continued on next page)

Exosomes, a subtype of $_S$EVs, are critical in shaping the tumor microenvironment.[16] This is particularly clear in the stromal compartment, where cancer-derived exosomes can activate fibroblasts through the transfer of molecules, such as transforming growth factor β (TGF-β).[16–19] Fibroblast activation leads to the deposition of extracellular matrix (ECM), tumor fibrosis, and metastasis.[20] This is particularly important in pancreatic cancer, where activation of the myofibroblast-like stellate cells and consequent fibrosis are the major contributors to the highly aggressive nature of these tumors and poor treatment efficacy.[21–23] Although some exosomal components are known to contribute to fibroblast activation and recruitment (e.g., TGF-β and Lin28B),[19,24] the pathways responsible for alterations in their packaging and secretion in cancer cells remain largely unknown.

Here, we show that the presence of extra centrosomes is sufficient to increase secretion of $_S$EVs, but not $_L$EVs. Characterization of these $_S$EVs by immunoelectron microscopy (IEM) and stable isotope labeling by amino acids in cell culture (SILAC) proteomic analyses suggests that they are of endocytic origin and thus enriched for exosomes. Mechanistically, we found that disruption of lysosome function, a consequence of increased ROS in cells with extra centrosomes, prevents efficient lysosome and multivesicular body fusion, leading to $_S$EV secretion. Furthermore, when compared to $_S$EVs secreted by pancreatic ductal adenocarcinoma (PDAC) cells with normal centrosome number, $_S$EVs secreted by cells with extra centrosomes are functionally distinct and can induce pancreatic stellate cell (PSC) activation. Consequently, PSCs pre-treated with $_S$EVs from cancer cells with extra centrosomes promote invasion of PDAC cells in heterotypic 3D cultures. Our findings demonstrate that centrosome amplification promotes quantitative and qualitative changes in secreted $_S$EVs that could influence communication between the tumor and the associated stroma to promote malignancy.

RESULTS

Centrosome amplification induces secretion of sEVs

Our previous work demonstrated that centrosome amplification leads to proteomic changes in the secretome, including an increase in proteins associated with EVs, suggesting higher EV secretion in cells with amplified centrosomes.[10] To explore this further, we used an established ultracentrifugation (UC) method[14] to crudely separate EVs according to their size: $_L$EVs and $_S$EVs, which we validated by nanoparticle tracking analyses (Figures S1A and S1B). To accurately measure secreted EV numbers, we used ImageStream flow cytometry to quantify fluorescently labeled EVs with the lipid dye BODIPY maleimide[25] and ensured that all serum was depleted for existing EVs by UC (Figures S1C and S1D). We found that, in the mammary epithelial cell line MCF10A, where we have previously performed secretome analysis,[10] induction of centrosome amplification by transient overexpression of the key regulator of centrosome duplication Polo-like kinase 4 (PLK4) in response to doxycycline (DOX)[26,27] led to increased secretion of $_S$EVs, but not $_L$EVs (Figure S1E).

Due to the well-established role of $_S$EVs in activating fibroblasts and its downstream effects on pancreatic cancer prognosis and treatment,[16,22] we decided to investigate whether the presence of extra centrosomes would impact $_S$EVs secretion in pancreatic cancer. To do this, we quantified the number of EVs and percentage of centrosome amplification in a panel of PDAC cell lines. For all experiments, the number of cells was optimized so that a similar cell number was obtained at the time EVs were collected (Table S1). We observed that cell lines with higher levels of centrosome amplification secreted increased numbers of EVs, in particular $_S$EVs, demonstrating a significant correlation between extra centrosomes and $_S$EV secretion (Figures 1A–1C and S1F). Furthermore, we confirmed that induction of centrosome amplification in two pancreatic cell lines, PaTu-S and HPAF-II, was sufficient to increase secretion of $_S$EVs, but not $_L$EVs (Figures 1D and S1G). Additionally, depletion of SAS-6, a protein important for centrosome duplication,[27] in cells exposed to DOX and PLK4 overexpression prevented both centrosome amplification and increased $_S$EV secretion but had no effect on $_S$EV secretion in control cells, suggesting that $_S$EV secretion is indeed a consequence of centrosomal alterations (Figures 1D and S1G–S1I).

The $_S$EVs fractions isolated by UC were enriched for several markers associated with exosomes, such as ALG-2 interacting protein-X (ALIX), CD63, tumor susceptibility gene 101 (TSG101), and CD81,[28] but not for general membrane markers, such as flotillin (Figure 1E). We further confirmed the presence of bona fide EVs in the $_S$EV fractions by electron microscopy (EM) and immunogold labeling using the $_S$EV marker CD63.[29] Consistent with increased $_S$EV secretion, we found that the percentage of CD63^{+ve} EVs was higher in cells with extra centrosomes (+DOX; Figure 1F). Moreover, these $_S$EVs were slightly larger, assessed by EM and also nanoparticle tracking analyses, suggesting that qualitative changes might also occur in these EVs (Figures 1G and S1J). Altogether, our results demonstrate that centrosome amplification promotes $_S$EV secretion.

Proteomic analyses of sEVs demonstrate their endocytic origin

To further understand the origin and composition of these $_S$EVs, we performed SILAC proteomic analyses.[30] SILAC labeling with

(B) Quantification of $_S$EVs and $_L$EVs secreted by PDAC cell lines. Average of the percentage of centrosome amplification (CA) per cell line is highlighted in orange.
(C) Linear regression of the data presented in (B) and Spearman correlation coefficients for $_S$EVs and $_L$EVs.
(D) Quantification of secreted $_S$EVs and $_L$EVs in PaTu-S.iPLK4 and HPAF-II.iPLK4 cell lines upon induction of centrosome amplification (+DOX), before and after depletion of Sas-6 by small interfering RNA (siRNA). Average percentage of CA per condition is highlighted in orange.
(E) Western blot analyses of proteins associated with $_S$EVs in extracts from cells and $_S$EVs collected by UC.
(F) Top: representative images of IEM of $_S$EVs collected from HPAF.iPLK4 cells. Dark beads represent immunogold labeling with anti-CD63. Scale bar, 200 nm. Bottom: quantification of the percentage of positive CD63 $_S$EVs is shown.
(G) Quantification of $_S$EVs diameter by cryoelectron microscopy (cryo-EM). PaTu-S.iPLK4 $_S$EVs $n_{(-DOX)} = 232$ and $n_{(+DOX)} = 216$; HPAF-II.iPLK4 $n_{(-DOX)} = 541$ and $n_{(+DOX)} = 493$.
For all graphics, error bars represent mean ± SD from three independent experiments. *p < 0.05, **p < 0.01, and ****p < 0.0001. The following statistics were applied: for graphs in (D), two-way ANOVA with Tukey's post hoc test was applied, and for graphs in (G), unpaired t test was applied. See also Figure S1 and Table S1.

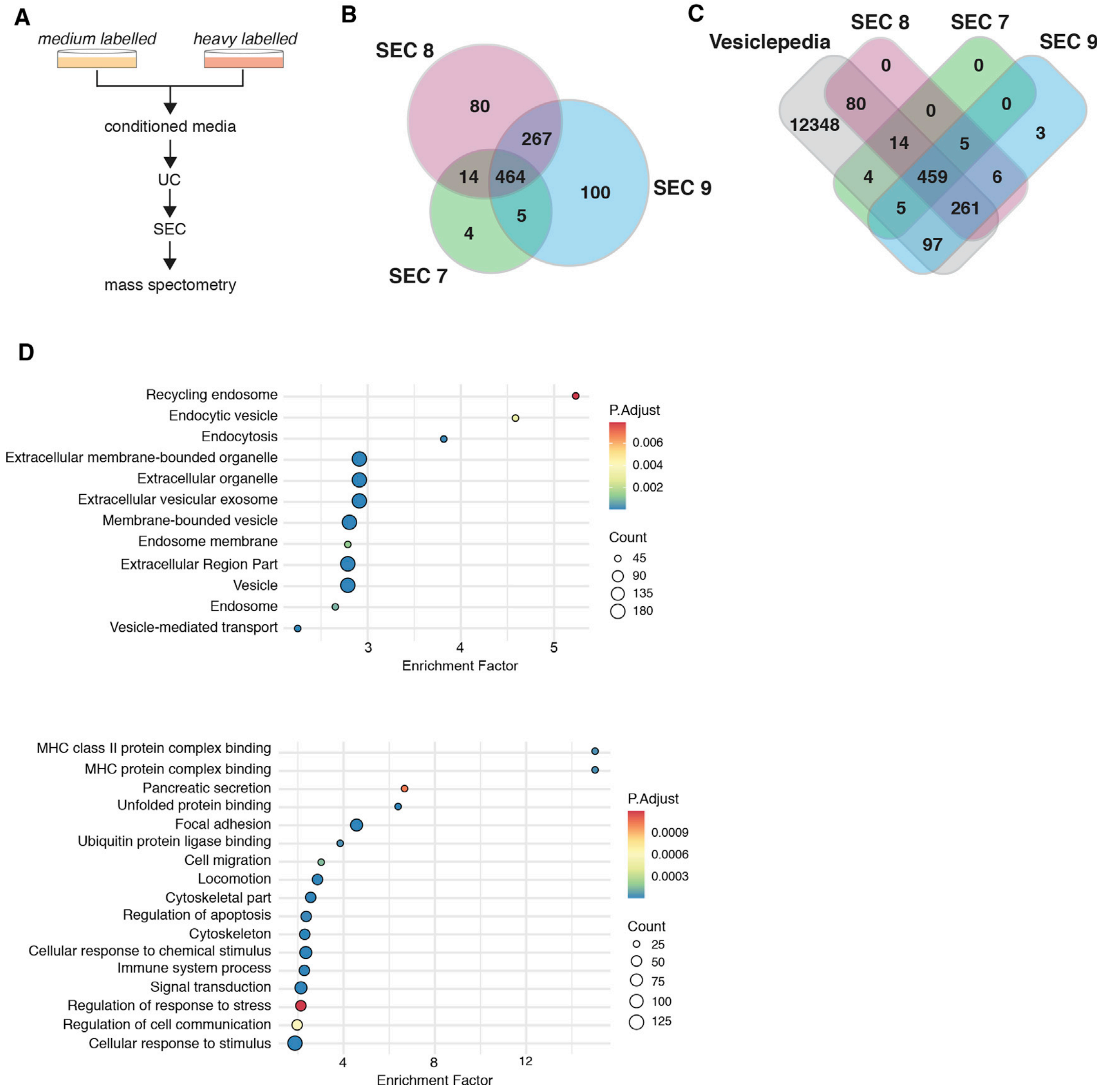

Figure 2. Proteomic analyses of sEVs secreted by cells with extra centrosomes support their endocytic origin

(A) Experimental flowchart.

(B) Venn diagram comparing the sEVs proteomes of SEC fractions 7–9.

(C) Venn diagram comparing the sEVs proteome of SEC fractions 7–9 with the Vesiclepedia database.

(D) Dot plot representation of the enrichment analyses performed for the common proteins in all SEC fractions. Only proteins that were identified in both forward and reverse labeling experiments were considered for this analysis.

See also Figure S2, Data S1, and Tables S2, S3, and S4.

medium and heavy isotopes enables the exclusion of contaminant serum proteins, which would be unlabeled (equivalent of light labeling), and allows for simultaneous processing of purification steps to decrease sample-to-sample variability (Figure 2A). Because UC isolated fractions can contain contaminants, such as protein aggregates and cellular debris, we further purified the sEVs UC fraction using size exclusion chromatography (SEC) prior to proteomics analysis (Figure S2A). Commercially available qEV SEC columns designed to purify exosomes were used,[31,32] and sEVs were quantified by ImageStream, as before. As expected for these columns, sEVs collected from PaTu-S.iPLK4 cells (±extra centrosomes) eluted in fractions 7–10, with the majority eluting in fractions 8 and 9 (Figure S2B). SILAC forward and reverse labeling was performed to conduct proteomic analyses of fractions 7–9. Quantitative analyses of the proteomic data for each SEC fraction revealed that approximately 464 proteins were common to all fractions and included known sEV components, such as ALIX, TSG101, CD81, and CD9 (Data S1A). There were also proteins unique to each fraction, suggesting that these sEVs are heterogeneous (Figure 2B; Data S1B–S1D). Comparison of our sEV proteomics data with the EV database Vesiclepedia[33] revealed that the majority of proteins in our datasets have been previously identified in other EV studies, confirming the robustness of our purification protocol.

Enrichment analyses of common proteins present in both SILAC forward and reverse labeling experiments were performed to identify common pathways (Tables S2 and S3). Importantly, the most significantly enriched categories were associated with EV and $_S$EV and linked to pathways unique for exosome biogenesis, such as recycling endosome and endocytic vesicles (Figure 2D). Moreover, pathways linked to cell communication, response to stress, pancreatic secretion, and immune response were also enriched in our dataset (Figure 2D), indicating that these $_S$EVs might have diverse functions.

To investigate whether centrosome amplification impacts on $_S$EV protein composition, we analyzed changes in the ratio of proteins present in heavy- and medium-labeled $_S$EV. Protein abundance was initially median normalized to ensure that heavy and medium intensities in each sample were equivalent. For proteins that a SILAC ratio could be calculated for, the ratio values did not significantly change in any SEC fraction (Figure S2C; Table S4). Interestingly, analyses of the original intensity profiles to determine whether total changes in protein were observed identified total loss/gain of 8 proteins in SEC fraction 7 and 6 proteins in SEC fraction 8, with the majority of changes being gains in $_S$EVs from cells with amplified centrosomes (Figures S2D and S2E). Thus, the presence of extra centrosomes does not induce a major change in the overall protein composition of $_S$EVs but instead affects the presence/absence of few specific proteins. Moreover, proteomic analyses of these $_S$EVs are consistent with an endocytic origin, indicating that this fraction is likely enriched for exosomes.

Impaired lysosomal function in cells with extra centrosomes promotes $_S$EV secretion

Multivesicular bodies are generally destined for degradation, by fusion with the lysosomal compartment, or are trafficked to the cell periphery, where they fuse with the plasma membrane, resulting in exosome secretion.[28,34] Lysosome dysfunction can shift the fate of multivesicular bodies targeted for degradation to fusion with plasma membrane, leading to increased $_S$EV secretion in non-transformed and cancer cells (Figure 3A).[35–37] We demonstrated previously that centrosome amplification increases ROS.[10] As ROS can disrupt lysosomal function,[38,39] we hypothesized that defective lysosomal degradation of multivesicular bodies could lead to increased $_S$EV secretion in cells with amplified centrosomes (Figure 3A). To test this, we first assessed whether induction of centrosome amplification led to increased ROS production in PDAC cell lines. Indeed, induction of extra centrosomes increased ROS in both PaTu-S.iPLK4 and HPAF-II.iPLK4 cell lines, as measured by the ratio of reduced (GSH) versus oxidized glutathione (GSSG), where a decrease indicates higher ROS levels (Figure 3B). Increased ROS can be abolished by treating cells with the ROS scavenger N-acetyl cysteine (NAC), while hydrogen peroxide (H_2O_2) is sufficient to increase ROS levels in these cells (Figures 3B and S3A). To determine lysosome functionality, we used Magic Red fluorescence intensity to assess the function of the lysosomal cathepsin B protease.[40] We found that cells with extra centrosomes have reduced Magic Red intensity levels and that treating cells with NAC prevented this defect, indicating that it is ROS dependent (Figures 3C, 3D, S3B, and S3F for examples of the original SUM intensity images used for this quantification). Furthermore, levels of lysosome-associated membrane glycoprotein-1 (LAMP1), a lysosomal marker, did not change in cells with extra centrosomes or in response to increased ROS (Figures S3C–S3E), suggesting that ROS specifically impair lysosome function, consistent with their role in disrupting the integrity of lysosomal membranes.[38] Note that, to account for changes in cell size in the different treatments, Magic Red and Lamp1 intensity levels were normalized to cell area. Next, we analyzed $_S$EV secretion in response to ROS. These analyses revealed that increased ROS was sufficient to increase $_S$EV secretion in PDAC cells and that preventing higher ROS production in cells with amplified centrosomes abolished enhanced $_S$EV secretion (Figure 3E). These results suggest that compromised lysosome function in cells with amplified centrosomes leads to $_S$EVs secretion. In agreement, inhibition of lysosome function with the vacuolar proton pump inhibitor bafilomycin A1, which impairs lysosome acidification,[41] was sufficient to increase $_S$EV secretion (Figures S3F–S3H).[42]

Next, we investigated whether ROS could prevent fusion between multivesicular bodies and lysosomes, thereby promoting multivesicular body fusion with the plasma membrane and, as a consequence, release of $_S$EVs (Figure 3A). Using an antibody against phospholipid lysobisphosphatidic acid (LBPA), a lipid enriched at the membranes of late endosomes and multivesicular bodies,[43] and lysotracker as a pH-based dye for functional lysosomes,[44] we quantified the co-localization of multivesicular bodies and lysosomes in the different conditions. Co-localization was quantified as the overlap between LBPA and lysotracker channels (Figure 4A, shown in white). Centrosome amplification decreased the number of lysotracker-positive intracellular vesicles in a ROS-dependent manner, but not LBPA-positive intracellular vesicles, further supporting defective lysosomal function as consequence of centrosome amplification (Figures 4A–4C). Strikingly, the percentage of co-localization between multivesicular bodies and lysosomes was significantly decreased in cells with extra centrosomes. NAC treatment restored lysosome function and multivesicular body-lysosome co-localization in cells with extra centrosomes, while H_2O_2 was sufficient to decrease co-localization (Figures 4A, 4B, and 4D). Furthermore, normalizing the co-localization data to total number of functional lysosomes abolished these differences (Figure 4E), demonstrating that reduced multivesicular body-lysosome co-localization is a direct result of decreased functional lysosomes in cells with amplified centrosomes or treated with H_2O_2. Consistently, impairing lysosome function with bafilomycin A1 dramatically reduced multivesicular body-lysosome co-localization (Figures S4A–S4E). Taken together, our data suggest that decreased multivesicular body-lysosome co-localization as a consequence of ROS-mediated lysosome dysfunction promotes $_S$EV secretion in cells with supernumerary centrosomes.

In cells with amplified centrosomes, decreased lysosomal-mediated multivesicular body degradation does not lead to increased multivesicular body numbers (Figure 4C). To determine whether decreased degradation of multivesicular bodies could instead result in changes in multivesicular body size, we quantified the size of LBPA vesicles. It has previously been described that the size of a multivesicular body is approximately 100–600 nm[45]; therefore, we only analyzed LBPA vesicles between 100 and 700 nm to prevent the quantification of small endosomes or LBPA aggregates. We did not find any obvious

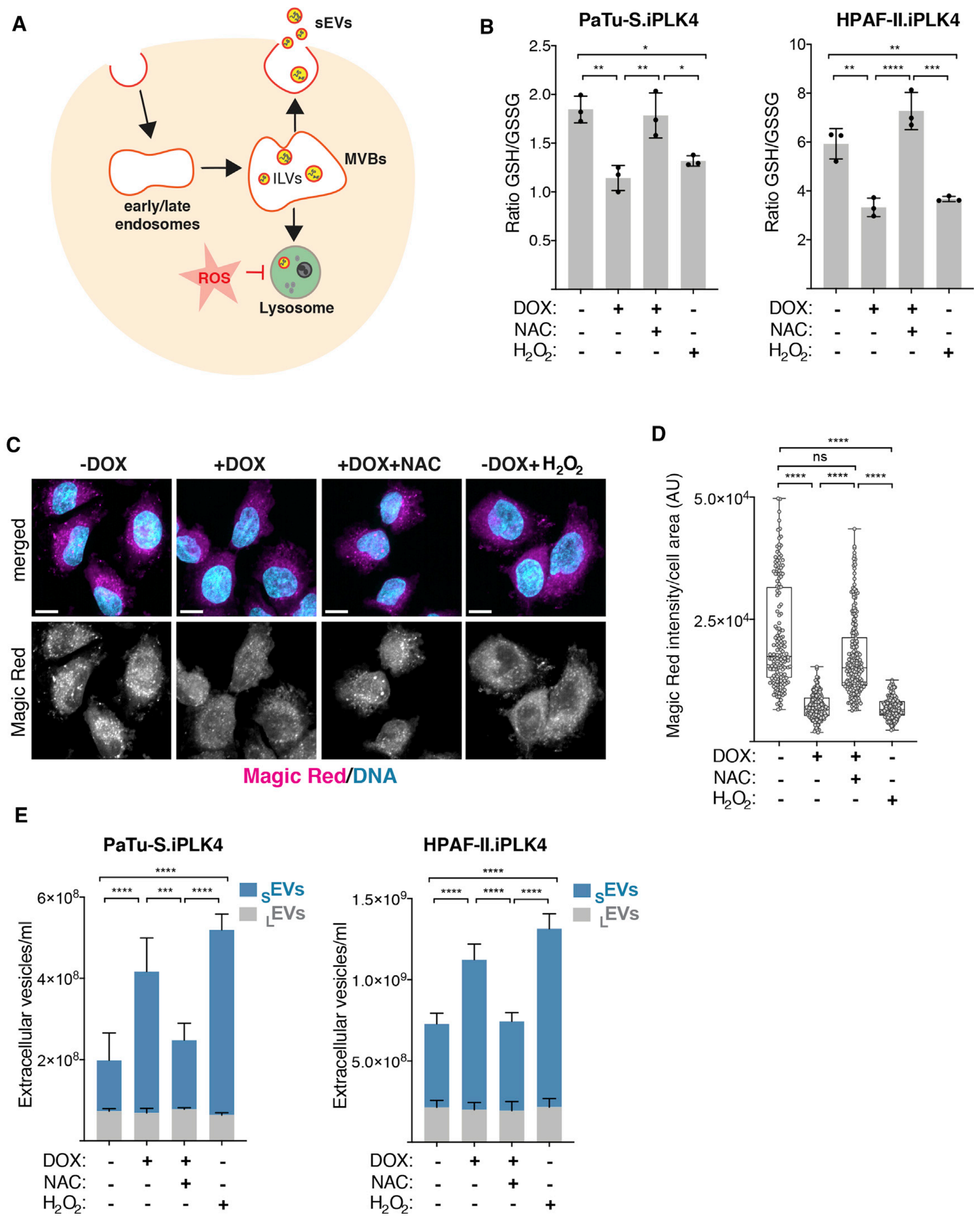

Figure 3. ROS promote lysosome dysfunction and $_S$EV secretion in cells with extra centrosomes

(A) Schematic representation of intraluminal vesicle formation (ILV) and multivesicular bodies (MVBs) fate and how ROS could affect this process.

(B) Levels of intracellular ROS quantified by the ratio of GSH/GSSG in PaTu-S.iPLK4 and HPAF-II.iPLK4 cell lines. Decrease in the GSH/GSSG ratio indicates higher ROS levels. 5 mM of NAC and 100 μM H_2O_2 were used.

(C) Representative confocal images of cells stained with Magic Red (magenta), as a proxy for lysosome function, and for DNA (cyan). MAX projection images shown (see Figure S3F for SUM intensity images). Scale bar, 10 μm.

(D) Quantification of intracellular Magic Red fluorescence intensity normalized for cell area in PaTu-S.iPLK4 cells. AU, arbitrary units. 5 mM of NAC and 100 μM H_2O_2 were used. $n_{(-DOX)} = 158$, $n_{(+DOX)} = 189$, $n_{(+DOX+NAC)} = 221$, and $n_{(-DOX+H2O2)} = 175$.

(E) Quantification of secreted $_S$EVs and $_L$EVs in PaTu-S.iPLK4 and HPAF-II.iPLK4 cell lines.

For all graphics, error bars represent mean ± SD from three independent experiments. *$p < 0.05$, **$p < 0.01$, ***$p < 0.001$, ****$p < 0.0001$, and n.s., not significant ($p > 0.05$). The following statistics were applied: for graphs in (B), one-way ANOVA with Tukey's post hoc test; for (D), one-way ANOVA with a Kruskal-Wallis post hoc test; and for (E) two-way ANOVA with Tukey's post hoc test. See also Figure S3.

CellPress
OPEN ACCESS

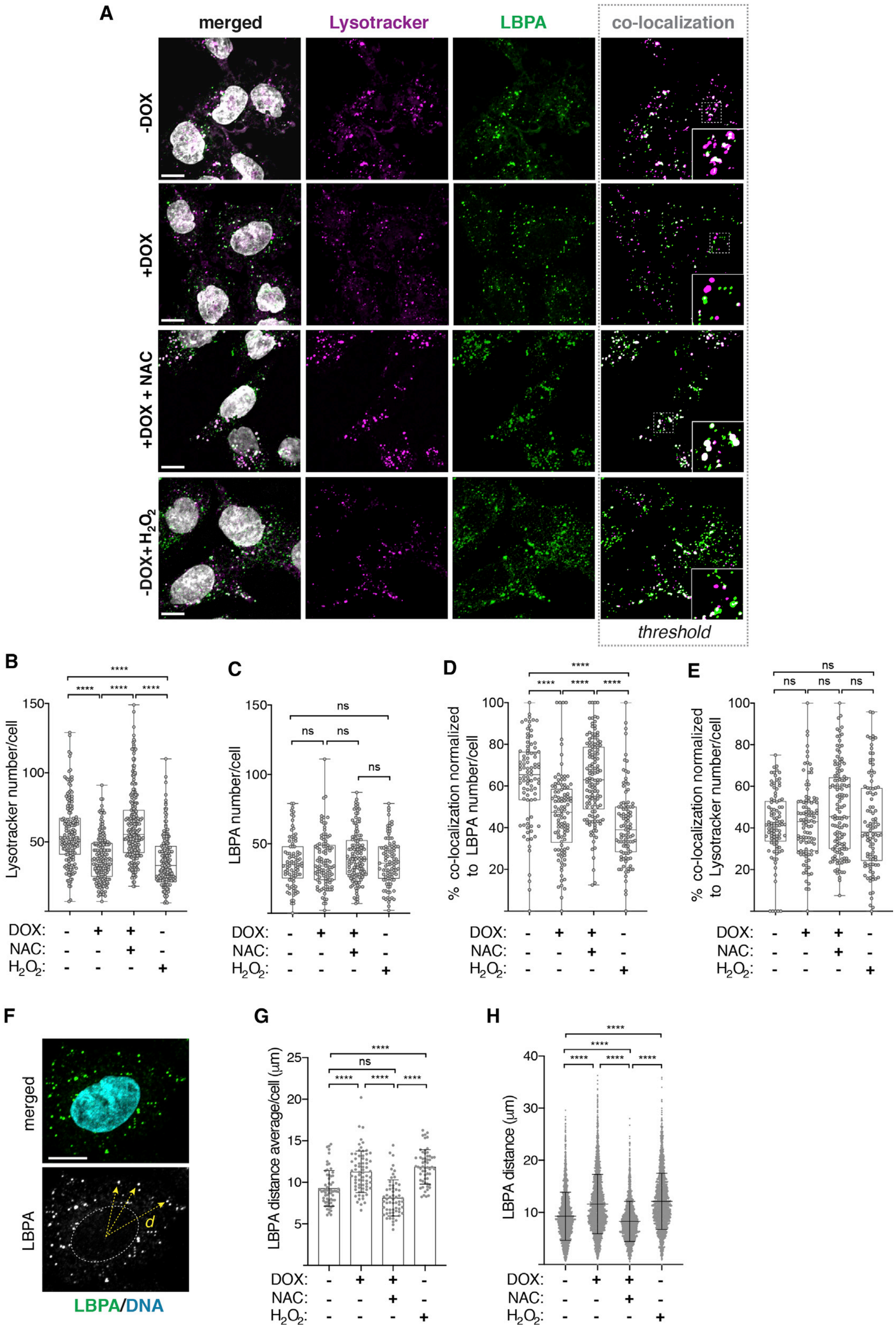

Figure 4. Centrosome amplification decreases MVBs-lysosome co-localization in a ROS-dependent manner

(A) Representative confocal images of cells stained for acidic lysosomes (lysotracker, magenta), late endosomes/MVBs (anti-LBPA, green), and DNA (gray). Insets show higher magnification of lysotracker and LBPA-labeled vesicles. Scale bar, 10 μm.

(legend continued on next page)

change in size of LBPA-labeled multivesicular bodies in the different conditions (Figures S4F and S4G). Next, we investigated whether trafficking of multivesicular bodies toward the cell periphery was altered by assessing the distance between LBPA vesicles and the center of the nucleus (Figure 4F). We observed that, in cells with extra centrosomes or those treated with H_2O_2, multivesicular bodies were dispersed toward the cell periphery (Figures 4G and 4H). Quantification of the distance between LBPA vesicles and the plasma membrane demonstrates that multivesicular bodies are closer to the plasma membrane in cells with extra centrosomes, in a ROS-dependent manner (Figures S4H and S4I). This effect became more obvious in the non-nuclear region of the cell, suggesting that multivesicular body trafficking towards the cell periphery could be associated with its proximity to the plasma membrane. Thus, these data suggest that, in addition to decreased multivesicular body degradation, changes in multivesicular bodies trafficking towards the plasma membrane could facilitate $_S$EV secretion in cells with extra centrosomes.[46]

sEVs secreted by cells with extra centrosomes activate pancreatic stellate cells to facilitate cancer cell invasion

Cancer-associated $_S$EVs often carry altered cargoes, rendering them functionally distinct from $_S$EVs secreted by non-transformed cells.[15,16] The exact causes of these changes, however, remain elusive. In PDAC, secreted $_S$EVs may contribute to fibrosis through the activation of PSCs.[47] Thus, we investigated whether $_S$EVs secreted by PDAC cells with extra centrosomes could promote the activation of PSCs. $_S$EVs collected from PDAC cells ± extra centrosomes (donor cells) were added to PSCs (Figure 5A). Equal numbers of $_S$EVs were added per condition to ensure that any differences observed were not due to the number of secreted $_S$EVs. Activation of PSCs was assessed by immunofluorescence of alpha smooth muscle actin (αSMA), with increased expression and association of αSMA with stress fibers as a common feature of PSC activation toward a myofibroblast-like phenotype (Figure 5B).[48] Interestingly, treatment of PSCs with $_S$EVs secreted by PDAC cells with extra centrosomes led to activation of ~25%–30% of the cell population (Figure 5C). It is important to note that, by normalizing $_S$EVs numbers, we are likely underestimating the differences between $_S$EVs secreted by cells ± centrosome amplification. As a positive control, PSCs were treated with TGF-β, a well-established activator of PSCs, known to lead to a strong activation phenotype (Figures S5A and S5B).[49]

To determine whether differential PSCs activation was due to changes in $_S$EV internalization, we quantified $_S$EV uptake. First, we show by immunofluorescence that labeled $_S$EVs clearly associate with PSCs (Figure 5D). Next, we used a recently described method where PSCs incubated with CellVue-labeled $_S$EVs are analyzed via ImageStream flow cytometry to quantify uptake (Figure S5C).[50] To control for any non-$_S$EV-mediated fluorescence, unstained cells and cells incubated with the condition medium used to label $_S$EVs with CellVue were also analyzed. We found that cells that had incorporated the labeled $_S$EVs could be easily identified (Figures 5E and S5D), and furthermore, quantification of these positive cells revealed no significant differences in the uptake of $_S$EVs secreted by cells with and without extra centrosomes (Figure 5F). Thus, differences in PSC activation are unlikely due to the differential uptake of $_S$EVs. Moreover, $_S$EVs collected from cells with and without extra centrosomes can slightly increase the proliferation of PSCs, as assessed by Ki67 (Figures S5D and S5E), highlighting that the functional differences between these $_S$EVs could be specific to PSC activation.

To further validate the PSC activation results, we purified the $_S$EVs by SEC (Figures S2B and S5F) and tested the activation potential of the different isolated fractions. Not only were the $_S$EVs harboring the potential to activate PSCs retained after SEC fractionation, but these $_S$EVs associated mainly with one fraction (SEC8 for PaTu-S.iPLK4 and SEC9 for HPAF-II.iPLK4; Figure 5G). Although the mechanism of PSCs activation of $_S$EVs secreted by cells with extra centrosomes is unclear, it is possible that differential loss/gain of proteins associated with SEC fraction 8 (Figure S2E) could play a role in this process.

Fibroblast activation is a common feature of cancer and can promote cancer cell invasion through various mechanisms, including ECM remodeling and proteolysis.[51] To determine the functional relevance of PSC activation by $_S$EVs secreted by PDAC cells with amplified centrosomes, we investigated their impact on PDAC cell invasion. To do so, we used 3D heterotypic cultures of HPAF-II cells that form spheroids in 3D with PSCs (Figure 6A).[52] In contrast to non-treated PSCs or PSCs pre-treated with $_S$EVs from cells with normal centrosome numbers, PSCs pre-treated with $_S$EVs harvested from cancer cells with extra centrosomes significantly induced invasion (Figures 6B and 6C). TGF-β pre-treated PSCs, used as positive control, showed higher invasion potential, consistent with the stronger levels of PSC activation observed (Figures 6B, 6C, and S5B). Confocal imaging of 3D spheroids composed of cancer cells expressing

(B) Quantification of the number of lysotracker-labeled lysosomes per cell. 5 mM of NAC and 100 μM H_2O_2 were used. $n_{(-DOX)} = 166$, $n_{(+DOX)} = 182$, $n_{(+DOX+NAC)} = 245$, and $n_{(-DOX+H2O2)} = 187$.
(C) Quantification of LBPA-labeled late endosomes/MVBs per cell. 5 mM of NAC and 100 μM H_2O_2 were used. $n_{(-DOX)} = 88$, $n_{(+DOX)} = 102$, $n_{(+DOX+NAC)} = 129$, and $n_{(-DOX+H2O2)} = x99$.
(D) Quantification of the percentage of lysotracker and LBPA-labeled intracellular vesicles co-localization normalized to LBPA numbers. 5 mM of NAC and 100 μM H_2O_2 were used. $n_{(-DOX)} = 86$, $n_{(+DOX)} = 102$, $n_{(+DOX+NAC)} = 129$, and $n_{(-DOX+H2O2)} = 98$.
(E) Quantification of the percentage of lysotracker and LBPA-labeled intracellular vesicles co-localization normalized to lysotracker number. 5 mM of NAC and 100 μM H_2O_2 were used. $n_{(-DOX)} = 86$, $n_{(+DOX)} = 102$, $n_{(+DOX+NAC)} = 129$, and $n_{(-DOX+H2O2)} = 98$.
(F) Representative image depicting method for quantifying LPBA distance from the nucleus center. Cells stained for LBPA (green) and DNA (cyan) are shown. Yellow arrows depict distance measured, *d*. Scale bar, 10 μm.
(G) Quantification of the average LBPA-nucleus center distance per cell. $n_{(-DOX)} = 62$, $n_{(+DOX)} = 68$, $n_{(+DOX+NAC)} = 61$, and $n_{(-DOX+H2O2)} = 57$.
(H) Quantification of all LBPA-nucleus center distance.
For all graphics, error bars represent mean ± SD from three independent experiments. *p < 0.05, **p < 0.01, ****p < 0.0001, and n.s. (p > 0.05). For all graphs, a one-way ANOVA with a Kruskal-Wallis post hoc test was applied. See also Figure S4.

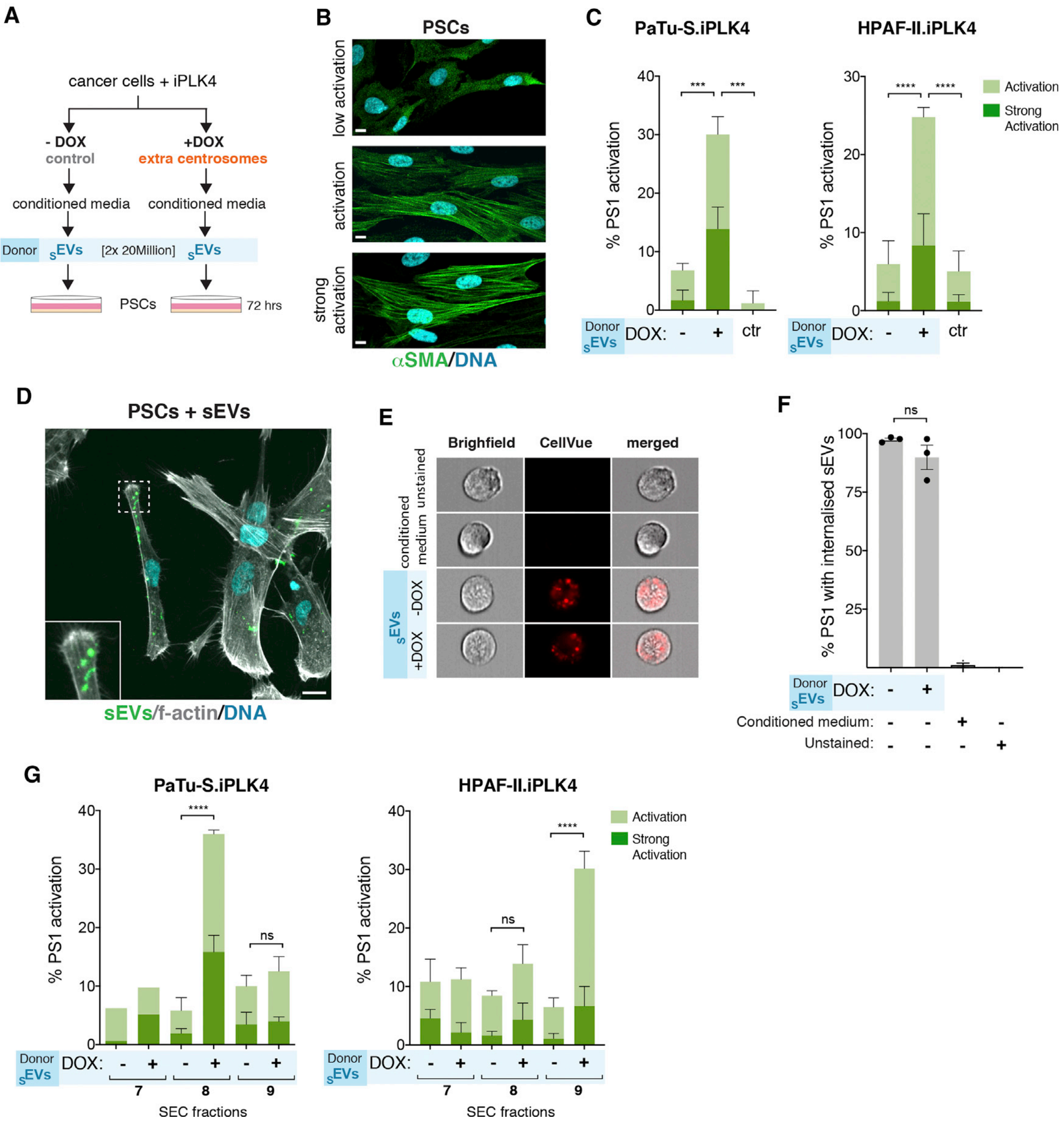

Figure 5. sEVs secreted by PDAC cells with amplified centrosomes activate pancreatic stellate cells

(A) Experimental flowchart.

(B) Representative confocal images of PSCs stained for αSMA (green) and DNA (cyan). Scale bar, 10 μm.

(C) Quantification of the percentage of PSCs activated upon treatment with sEVs collected by UC from PaTu-S.iPLK4 (left) and HPAF-II.iPLK4 (right), with (+DOX) and without (−DOX) extra centrosomes. PaTu-S.iPLK4 isolated sEV: PSCs $n_{(-DOX\ sEVs)} = 398$; $n_{(+DOX\ sEVs)} = 373$; and $n_{(ctr)} = 475$. HPAF-II.iPLK4 isolated sEV: PSCs $n_{(-DOX\ sEVs)} = 914$; $n_{(+DOX\ sEVs)} = 1{,}057$; and $n_{(ctr)} = 718$.

(D) Representative confocal image of PSCs incubated with sEVs. Cells were stained for f-actin (phalloidin, gray) and DNA (cyan). Isolated sEVs were labeled with BODIPY (green). Inset depicts higher magnification of sEVs associated with PSCs. Scale bar, 10 μm.

(E) Representative images of cells acquired with the ImageStream. Cells (gray, bright field) and internalized sEV labeled with CellVue (red) are shown.

(F) Quantification of the percentage of PS1 cells positive for CellVue labeling. $n_{(unstained)} = 6{,}280$, $n_{(cond.\ medium)} = 6{,}417$, $n_{(-DOX\ sEVs)} = 7{,}066$, and $n_{(+DOX\ sEVs)} = 7{,}230$.

(G) Quantification of the percentage of PSCs activated upon treatment with sEVs collected by UC followed by SEC from PaTu-S.iPLK4 (left) and HPAF-II.iPLK4 (right), with (+DOX) and without (−DOX) extra centrosomes. PaTu-S.iPLK4 isolated sEV: PSCs $n_{(-DOX\ sEVs\ SEC7)} = 161$; $n_{(+DOX\ sEVs\ SEC7)} = 154$; PSCs $n_{(-DOX\ sEVs\ SEC8)} = 490$; $n_{(+DOX\ sEVs\ SEC8)} = 387$; PSCs $n_{(-DOX\ sEVs\ SEC9)} = 463$; and $n_{(+DOX\ sEVs\ SEC7)} = 454$. HPAF-II.iPLK4 isolated sEV: PSCs $n_{(-DOX\ sEVs\ SEC7)} = 499$; $n_{(+DOX\ sEVs\ SEC7)} = 410$; PSCs $n_{(-DOX\ sEVs\ SEC8)} = 541$; $n_{(+DOX\ sEVs\ SEC8)} = 713$; PSCs $n_{(-DOX\ sEVs\ SEC9)} = 1{,}035$; and $n_{(+DOX\ sEVs\ SEC7)} = 914$.

For all graphics, error bars represent mean ± SD from three independent experiments. ***$p < 0.001$, ****$p < 0.0001$, and n.s. ($p > 0.05$). For all, graphs were analyzed using two-way ANOVA with Tukey's post hoc test. See also Figure S5.

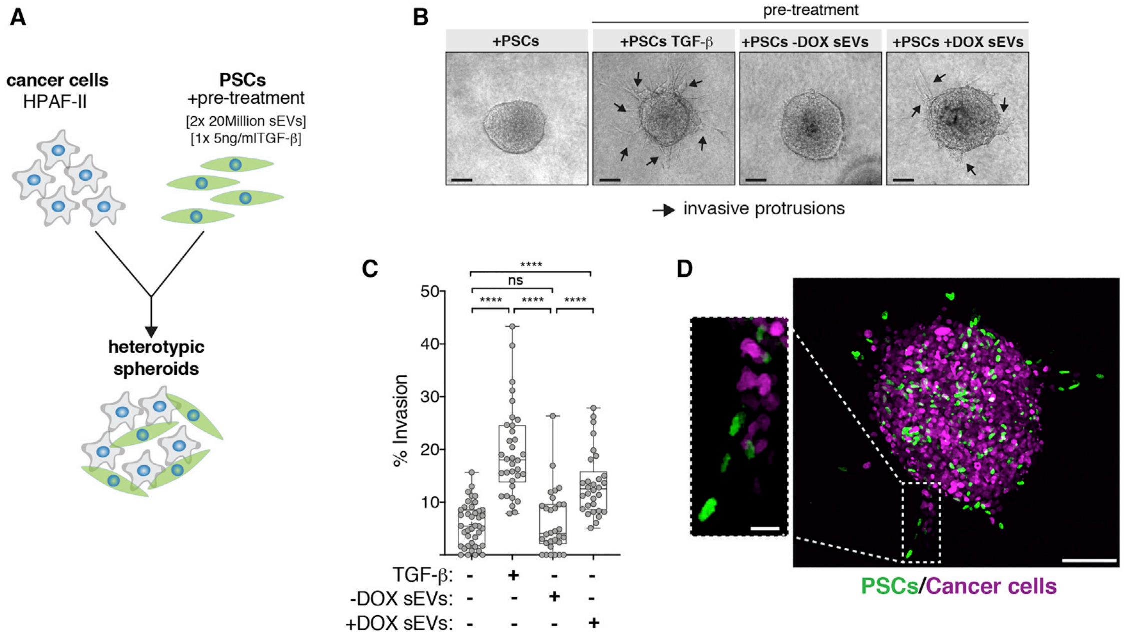

Figure 6. $_{S}$EVs secreted by cells with extra centrosomes can promote PDAC invasion

(A) Experimental flowchart.

(B) Representative bright-field images of heterotypic spheroids. Black arrows: invasive protrusions. Scale bar, 100 μm.

(C) Quantification of the percentage of invasion in 3D spheroids. 5 ng/mL TGF-β was used as positive control. Spheroids $n_{(+PSCs)} = 40$, $n_{(+PSCs\ TGF\text{-}\beta)} = 34$, $n_{(+PSCs\ -DOX\ SEVs)} = 31$, and $n_{(+PSCs\ +DOX\ SEVs)} = 31$.

(D) Confocal images of spheroids composed of cancer cells (expressing H_2B-RFP; magenta) and PSCs (expressing H_2B-GFP; green). Scale bar, 100 μm. Inset depicts higher magnification of invasive protrusion. Scale bar, 20 μm.

For all graphics, error bars represent mean ± SD from three independent experiments. ****$p < 0.0001$, n.s. ($p > 0.05$). Graph was analyzed using one-way ANOVA with a Kruskal-Wallis post hoc test.

H_2B-RFP and PSCs expressing H_2B-GFP revealed that activated PSCs lead the invasive front (Figure 6D). Our findings demonstrate that sEVs secreted by PDAC cells with extra centrosomes are functionally different and can induce PSCs activation to promote cancer invasion.

DISCUSSION

In this study, we demonstrate that centrosome amplification induces secretion of $_{S}$EVs that activate PSCs, which in turn promote the invasion of cancer spheroids. Activated PSCs are major players in the development of the pancreatic cancer stroma and associated fibrosis,[21–23] suggesting a role for centrosome amplification in shaping the pancreatic cancer microenvironment. Our data support a model whereby elevated ROS levels induced by extra centrosomes lead to loss of lysosomal function, favoring multivesicular bodies fusion with the plasma membrane and $_{S}$EV secretion (Figure 7).

Lysosomes are signaling centers that integrate many cellular responses to changes in nutrients, growth factors, and stresses.[53] Fusion of lysosomes with autophagosomes is critical during autophagy, a self-degradative process important for the removal of protein aggregates, damaged organelles, and intracellular pathogens.[53] Centrosome amplification was recently shown to disrupt autophagy, rendering these cells sensitive to autophagy inhibitors.[54] We currently do not know whether lysosome dysfunction is responsible for the autophagy defects observed in these cells. However, it is reasonable to assume that ROS-mediated lysosomal dysregulation could have a broader impact on the physiology of cells carrying centrosomal abnormalities.

The fate of multivesicular bodies is also determined by its transport along the microtubules, which depends on the Rab family of guanosine triphosphatases (GTPases) and molecular motors, such as kinesins.[55] Indeed, plus-end directed motors, such as members of the kinesin-1 family, are important to transport late endosomes and multivesicular bodies to the plasma membrane.[46] We found that LBPA-labeled multivesicular bodies are dispersed toward the cell periphery and closer to the plasma membrane in cells with amplified centrosomes or that have been treated with H_2O_2. These observations are consistent with kinesin-1-driven transport that could facilitate $_{S}$EV release. Thus, it is possible that, in addition to defective lysosome function, changes in the microtubules and/or associated motors could play a role in $_{S}$EV secretion in response to centrosome amplification and increased ROS.

$_{S}$EVs secreted by cells with extra centrosomes exhibit many characteristics of exosomes: correct size range (30–150 nm) and proteomic profiling revealed an enrichment for proteins associated with exosomes and exosome biogenesis. Sub-fractionation of secreted $_{S}$EVs by SEC demonstrated not only the existence of different subtypes of $_{S}$EVs, as previously reported,[56,57] but that functional differences between these different $_{S}$EV populations also exist, as assessed by their ability to activate stellate cells.

CellPress
OPEN ACCESS

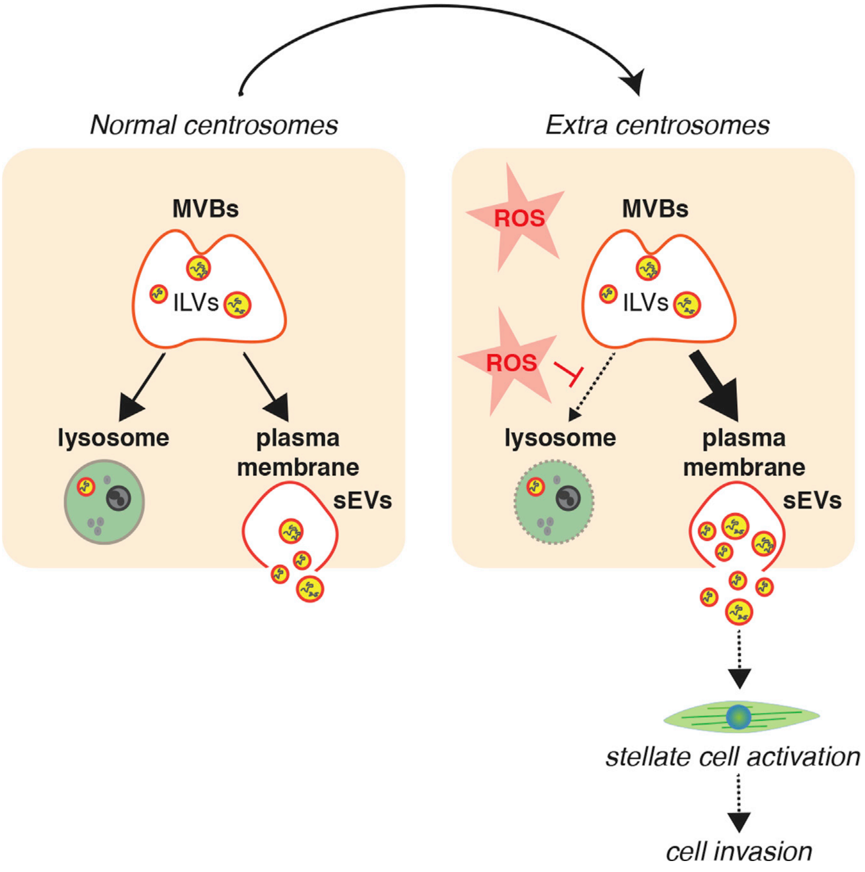

Figure 7. Schematic representation of working model

Increased ROS levels in cells with extra centrosomes compromise lysosomal function. We propose that this changes MVBs fate toward fusing with the plasma membrane, resulting in increased secretion of sEVs. sEVs secreted by cancer cells with extra centrosomes are functionally distinct and can induce PSCs activation to promote cell invasion.

How changes in sEV composition occur and how these induce stellate cell activation, however, remains elusive. One possibility is that changes in the sEV cargoes (proteins and RNA species) could be involved in stellate cell activation. Indeed, although SILAC ratio values for most detected proteins remained unchanged, we identified a number of proteins that were only identified in one label, for which a SILAC ratio could not be calculated. In particular, we identified five proteins that were present in the sEV fraction that activate PSCs: phosphoglucomutase 3 (PGM3); carbamoyl-phosphate synthetase 2 aspartate transcarbamylase and dihydroorotase (CAD); mitochondria encoded cytochrome C oxidase II (MT-CO2); FAM129A (NIBAN); and coiled-coil domain containing 124 (CCDC214). Therefore, it is possible that some of these proteins could play a role in PSC activation, but further studies will be required to assess whether this is the case.

Alternatively, the presence or absence of specific proteins could influence sEV uptake and/or intracellular fate and indirectly contribute to PSC activation. Cargo transfer by EVs can be mediated by delivery of surface proteins to membrane receptors, fusion with the plasma membrane, micropinocytosis, phagocytosis, and receptor-mediated endocytosis to deliver their content.[58] In addition, interaction between EVs and secreted proteins has been shown to modulate their uptake, highlighting the complex regulation of this process.[59] Tetraspanins, such as CD9, CD63, and CD81, have been shown to be involved in the interplay between adhesion molecules and integrins to promote sEV uptake.[60] The presence of specific tetraspanins could also influence the specificity of target cells. For example, sEVs lacking the expression of the tetraspanin CD63 were found to be preferentially endocytosed by neurons.[61] We found that CD81 was the only protein absent specifically in the sEVs harvested from PDAC cells with amplified centrosomes that activate PSCs. Although our data suggest that the global uptake of sEVs secreted from cells with and without extra centrosomes is equally efficient, we cannot rule out that loss of CD81 could play a role in cargo transfer or intracellular fate of sEVs. Interestingly, loss of CD81 has previously been reported in sEVs that are secreted upon induction of lysosome dysfunction.[36] The reason for CD81 loss in response to lysosomal dysregulation is currently unknown, but the striking similarity suggests a common response to lysosomal defects that could potentially modulate sEV uptake and/or intracellular fate.

In summary, we describe a mechanism by which a stress response downstream of extra centrosomes culminates with the secretion of functionally distinct sEVs by diverging the fate of multivesicular bodies. Several cellular stresses have been shown to induce EV secretion, such as oxidative stress, hypoxia, and radiation-induced cell stress.[62] Thus, it is possible that, in response to multiple stressors, multivesicular bodies that are normally targeted for lysosomal degradation play a role in the release of sEVs carrying protective functions in order to maintain tissue homeostasis. Indeed, oxidative stress itself has been shown to induce changes in the mRNA content of exosomes secreted by mouse mast cells, which help to protect the surrounding cells by conferring resistance to subsequent oxidative insult.[63] Understanding how stress communication protects cancer cells could allow us to exploit these mechanisms to prevent cancer cell adaptation.

STAR★METHODS

Detailed methods are provided in the online version of this paper and include the following:

- KEY RESOURCES TABLE
- RESOURCE AVAILABILITY

- Lead contact
- Material availability
- Data and code availability
- EXPERIMENTAL MODEL AND SUBJECT DETAILS
  - Cell lines and culture conditions
- METHOD DETAILS
  - Chemicals
  - Lentiviral production and Infection
  - siRNA
  - Immunofluorescence 2D
  - Immunofluorescence data analyzes
  - Extracellular Vesicle Quantification and Analysis
  - Extracellular vesicle uptake by recipient cells
- QUANTIFICATION AND STATISTICAL ANALYSIS
  - Statistical analysis

SUPPLEMENTAL INFORMATION

Supplemental Information can be found online at https://doi.org/10.1016/j.cub.2021.01.028.

ACKNOWLEDGMENTS

We are grateful to Judith Simon, Sam Wallis, and all the members of the Godinho lab for comments or discussion of the manuscript; Andrea Lafuente for providing the dot plots used in Figure 2D; Hefin Rhys for helping with ImageStream and Nanosight; and Pedro Monteiro for advice on image analysis. This work was supported by a Cancer Research UK Centre Grant to Barts Cancer Institute (C355/A25137). E.P.C. and R.G. were funded by Cancer Research UK (C10847/A27781). G.R. is supported by the French government (ANR) through the 20 Investments for the Future LABEX SIGNALIFE (ANR-11-LABX-0028-01) and by the Fondation pour la Recherche Medicale (DEQ2011104211324). F.M. is supported by a Medical Research Council (MRC) Career Development Award (MR/P009417/1) and a Barts Charity grant (MGU0346). S.D.A. was supported by an MRC PhD studentship and J.C. supported by a Barry Reed PhD studentship. S.A.G. is a fellow of the Lister Institute and is supported by the MRC (MR/T000538/1).

AUTHOR CONTRIBUTIONS

Conceptualization, S.D.A. and S.A.G.; Methodology, S.D.A., J.C., T.A., G.D., E.P.C., M.R., R.G., G.R., F.M., and S.A.G.; Validation, S.D.A. and J.C.; Formal Analysis, S.D.A., J.C., G.D., F.M., and S.A.G.; Investigation, S.D.A., J.C., T.A., G.D., E.P.C., and M.D.; Resources, H.M.K., R.G., G.R., F.M., and S.A.G.; Data Curation, S.D.A., J.C., and F.M.; Writing – Original Draft, S.A.G.; Writing – Review & Editing, S.D.A., G.D., E.P.C., H.M.K., R.G., G.R., F.M., and S.A.G.; Visualization, S.D.A., J.C., G.D., E.P.C., and S.A.G.; Supervision, H.M.K., G.R., F.M., and S.A.G.; Project Administration, S.A.G.; Funding Acquisition, S.A.G.

DECLARATION OF INTERESTS

The authors declare no competing interests.

Received: August 20, 2020
Revised: December 1, 2020
Accepted: January 8, 2021
Published: February 15, 2021

REFERENCES

1. Chan, J.Y. (2011). A clinical overview of centrosome amplification in human cancers. Int. J. Biol. Sci. *7*, 1122–1144.
2. Zyss, D., and Gergely, F. (2009). Centrosome function in cancer: guilty or innocent? Trends Cell Biol. *19*, 334–346.
3. Basto, R., Brunk, K., Vinadogrova, T., Peel, N., Franz, A., Khodjakov, A., and Raff, J.W. (2008). Centrosome amplification can initiate tumorigenesis in flies. Cell *133*, 1032–1042.
4. Levine, M.S., Bakker, B., Boeckx, B., Moyett, J., Lu, J., Vitre, B., Spierings, D.C., Lansdorp, P.M., Cleveland, D.W., Lambrechts, D., et al. (2017). Centrosome amplification is sufficient to promote spontaneous tumorigenesis in mammals. Dev. Cell *40*, 313–322.e5.
5. Coelho, P.A., Bury, L., Shahbazi, M.N., Liakath-Ali, K., Tate, P.H., Wormald, S., Hindley, C.J., Huch, M., Archer, J., Skarnes, W.C., et al. (2015). Over-expression of Plk4 induces centrosome amplification, loss of primary cilia and associated tissue hyperplasia in the mouse. Open Biol. *5*, 150209.
6. Serçin, Ö., Larsimont, J.C., Karambelas, A.E., Marthiens, V., Moers, V., Boeckx, B., Le Mercier, M., Lambrechts, D., Basto, R., and Blanpain, C. (2016). Transient PLK4 overexpression accelerates tumorigenesis in p53-deficient epidermis. Nat. Cell Biol. *18*, 100–110.
7. Ganem, N.J., Godinho, S.A., and Pellman, D. (2009). A mechanism linking extra centrosomes to chromosomal instability. Nature *460*, 278–282.
8. Godinho, S.A., and Pellman, D. (2014). Causes and consequences of centrosome abnormalities in cancer. Philos. Trans. R. Soc. Lond. B Biol. Sci. *369*, 20130467.
9. Silkworth, W.T., Nardi, I.K., Scholl, L.M., and Cimini, D. (2009). Multipolar spindle pole coalescence is a major source of kinetochore mis-attachment and chromosome mis-segregation in cancer cells. PLoS ONE *4*, e6564.
10. Arnandis, T., Monteiro, P., Adams, S.D., Bridgeman, V.L., Rajeeve, V., Gadaleta, E., Marzec, J., Chelala, C., Malanchi, I., Cutillas, P.R., and Godinho, S.A. (2018). Oxidative stress in cells with extra centrosomes drives non-cell-autonomous invasion. Dev. Cell *47*, 409–424.e9.
11. Place, A.E., Jin Huh, S., and Polyak, K. (2011). The microenvironment in breast cancer progression: biology and implications for treatment. Breast Cancer Res. *13*, 227.
12. Quail, D.F., and Joyce, J.A. (2013). Microenvironmental regulation of tumor progression and metastasis. Nat. Med. *19*, 1423–1437.
13. van Niel, G., D'Angelo, G., and Raposo, G. (2018). Shedding light on the cell biology of extracellular vesicles. Nat. Rev. Mol. Cell Biol. *19*, 213–228.
14. Costa-Silva, B., Aiello, N.M., Ocean, A.J., Singh, S., Zhang, H., Thakur, B.K., Becker, A., Hoshino, A., Mark, M.T., Molina, H., et al. (2015). Pancreatic cancer exosomes initiate pre-metastatic niche formation in the liver. Nat. Cell Biol. *17*, 816–826.
15. Hurwitz, S.N., Rider, M.A., Bundy, J.L., Liu, X., Singh, R.K., and Meckes, D.G., Jr. (2016). Proteomic profiling of NCI-60 extracellular vesicles uncovers common protein cargo and cancer type-specific biomarkers. Oncotarget *7*, 86999–87015.
16. Xu, R., Rai, A., Chen, M., Suwakulsiri, W., Greening, D.W., and Simpson, R.J. (2018). Extracellular vesicles in cancer - implications for future improvements in cancer care. Nat. Rev. Clin. Oncol. *15*, 617–638.
17. Webber, J., Steadman, R., Mason, M.D., Tabi, Z., and Clayton, A. (2010). Cancer exosomes trigger fibroblast to myofibroblast differentiation. Cancer Res. *70*, 9621–9630.
18. Webber, J.P., Spary, L.K., Sanders, A.J., Chowdhury, R., Jiang, W.G., Steadman, R., Wymant, J., Jones, A.T., Kynaston, H., Mason, M.D., et al. (2015). Differentiation of tumour-promoting stromal myofibroblasts by cancer exosomes. Oncogene *34*, 290–302.
19. Ringuette Goulet, C., Bernard, G., Tremblay, S., Chabaud, S., Bolduc, S., and Pouliot, F. (2018). Exosomes induce fibroblast differentiation into cancer-associated fibroblasts through TGFβ signaling. Mol. Cancer Res. *16*, 1196–1204.
20. Mohammadi, H., and Sahai, E. (2018). Mechanisms and impact of altered tumour mechanics. Nat. Cell Biol. *20*, 766–774.
21. Fujita, H., Ohuchida, K., Mizumoto, K., Nakata, K., Yu, J., Kayashima, T., Cui, L., Manabe, T., Ohtsuka, T., and Tanaka, M. (2010). alpha-smooth muscle actin expressing stroma promotes an aggressive tumor biology in pancreatic ductal adenocarcinoma. Pancreas *39*, 1254–1262.

CellPress
OPEN ACCESS

22. McCarroll, J.A., Naim, S., Sharbeen, G., Russia, N., Lee, J., Kavallaris, M., Goldstein, D., and Phillips, P.A. (2014). Role of pancreatic stellate cells in chemoresistance in pancreatic cancer. Front. Physiol. *5*, 141.

23. Kadaba, R., Birke, H., Wang, J., Hooper, S., Andl, C.D., Di Maggio, F., Soylu, E., Ghallab, M., Bor, D., Froeling, F.E., et al. (2013). Imbalance of desmoplastic stromal cell numbers drives aggressive cancer processes. J. Pathol. *230*, 107–117.

24. Zhang, Y.F., Zhou, Y.Z., Zhang, B., Huang, S.F., Li, P.P., He, X.M., Cao, G.D., Kang, M.X., Dong, X., and Wu, Y.L. (2019). Pancreatic cancer-derived exosomes promoted pancreatic stellate cells recruitment by pancreatic cancer. J. Cancer *10*, 4397–4407.

25. Headland, S.E., Jones, H.R., D'Sa, A.S., Perretti, M., and Norling, L.V. (2014). Cutting-edge analysis of extracellular microparticles using ImageStream(X) imaging flow cytometry. Sci. Rep. *4*, 5237.

26. Godinho, S.A., Picone, R., Burute, M., Dagher, R., Su, Y., Leung, C.T., Polyak, K., Brugge, J.S., Théry, M., and Pellman, D. (2014). Oncogene-like induction of cellular invasion from centrosome amplification. Nature *510*, 167–171.

27. Nigg, E.A., and Holland, A.J. (2018). Once and only once: mechanisms of centriole duplication and their deregulation in disease. Nat. Rev. Mol. Cell Biol. *19*, 297–312.

28. Kowal, J., Tkach, M., and Théry, C. (2014). Biogenesis and secretion of exosomes. Curr. Opin. Cell Biol. *29*, 116–125.

29. Colombo, M., Moita, C., van Niel, G., Kowal, J., Vigneron, J., Benaroch, P., Manel, N., Moita, L.F., Théry, C., and Raposo, G. (2013). Analysis of ESCRT functions in exosome biogenesis, composition and secretion highlights the heterogeneity of extracellular vesicles. J. Cell Sci. *126*, 5553–5565.

30. Mardakheh, F.K., Sailem, H.Z., Kümper, S., Tape, C.J., McCully, R.R., Paul, A., Anjomani-Virmouni, S., Jørgensen, C., Poulogiannis, G., Marshall, C.J., and Bakal, C. (2016). Proteomics profiling of interactome dynamics by colocalisation analysis (COLA). Mol. Biosyst. *13*, 92–105.

31. Böing, A.N., van der Pol, E., Grootemaat, A.E., Coumans, F.A., Sturk, A., and Nieuwland, R. (2014). Single-step isolation of extracellular vesicles by size-exclusion chromatography. J. Extracell. Vesicles *3*, https://doi.org/10.3402/jev.v3.23430.

32. Muller, L., Hong, C.S., Stolz, D.B., Watkins, S.C., and Whiteside, T.L. (2014). Isolation of biologically-active exosomes from human plasma. J. Immunol. Methods *411*, 55–65.

33. Kalra, H., Simpson, R.J., Ji, H., Aikawa, E., Altevogt, P., Askenase, P., Bond, V.C., Borràs, F.E., Breakefield, X., Budnik, V., et al. (2012). Vesiclepedia: a compendium for extracellular vesicles with continuous community annotation. PLoS Biol. *10*, e1001450.

34. Harding, C., Heuser, J., and Stahl, P. (1983). Receptor-mediated endocytosis of transferrin and recycling of the transferrin receptor in rat reticulocytes. J. Cell Biol. *97*, 329–339.

35. Alvarez-Erviti, L., Seow, Y., Schapira, A.H., Gardiner, C., Sargent, I.L., Wood, M.J., and Cooper, J.M. (2011). Lysosomal dysfunction increases exosome-mediated alpha-synuclein release and transmission. Neurobiol. Dis. *42*, 360–367.

36. Latifkar, A., Ling, L., Hingorani, A., Johansen, E., Clement, A., Zhang, X., Hartman, J., Fischbach, C., Lin, H., Cerione, R.A., and Antonyak, M.A. (2019). Loss of Sirtuin 1 alters the secretome of breast cancer cells by impairing lysosomal integrity. Dev. Cell *49*, 393–408.e7.

37. Miao, Y., Li, G., Zhang, X., Xu, H., and Abraham, S.N. (2015). A TRP channel senses lysosome neutralization by pathogens to trigger their expulsion. Cell *161*, 1306–1319.

38. Aits, S., and Jäättelä, M. (2013). Lysosomal cell death at a glance. J. Cell Sci. *126*, 1905–1912.

39. Nilsson, E., Ghassemifar, R., and Brunk, U.T. (1997). Lysosomal heterogeneity between and within cells with respect to resistance against oxidative stress. Histochem. J. *29*, 857–865.

40. Leung-Toung, R., Li, W., Tam, T.F., and Karimian, K. (2002). Thiol-dependent enzymes and their inhibitors: a review. Curr. Med. Chem. *9*, 979–1002.

41. Yoshimori, T., Yamamoto, A., Moriyama, Y., Futai, M., and Tashiro, Y. (1991). Bafilomycin A1, a specific inhibitor of vacuolar-type H(+)-ATPase, inhibits acidification and protein degradation in lysosomes of cultured cells. J. Biol. Chem. *266*, 17707–17712.

42. Savina, A., Furlán, M., Vidal, M., and Colombo, M.I. (2003). Exosome release is regulated by a calcium-dependent mechanism in K562 cells. J. Biol. Chem. *278*, 20083–20090.

43. Kobayashi, T., Stang, E., Fang, K.S., de Moerloose, P., Parton, R.G., and Gruenberg, J. (1998). A lipid associated with the antiphospholipid syndrome regulates endosome structure and function. Nature *392*, 193–197.

44. Zhitomirsky, B., Farber, H., and Assaraf, Y.G. (2018). LysoTracker and MitoTracker Red are transport substrates of P-glycoprotein: implications for anticancer drug design evading multidrug resistance. J. Cell. Mol. Med. *22*, 2131–2141.

45. Hanson, P.I., and Cashikar, A. (2012). Multivesicular body morphogenesis. Annu. Rev. Cell Dev. Biol. *28*, 337–362.

46. Bonifacino, J.S., and Neefjes, J. (2017). Moving and positioning the endolysosomal system. Curr. Opin. Cell Biol. *47*, 1–8.

47. Masamune, A., Yoshida, N., Hamada, S., Takikawa, T., Nabeshima, T., and Shimosegawa, T. (2018). Exosomes derived from pancreatic cancer cells induce activation and profibrogenic activities in pancreatic stellate cells. Biochem. Biophys. Res. Commun. *495*, 71–77.

48. Erkan, M., Adler, G., Apte, M.V., Bachem, M.G., Buchholz, M., Detlefsen, S., Esposito, I., Friess, H., Gress, T.M., Habisch, H.J., et al. (2012). StellaTUM: current consensus and discussion on pancreatic stellate cell research. Gut *61*, 172–178.

49. Apte, M.V., Park, S., Phillips, P.A., Santucci, N., Goldstein, D., Kumar, R.K., Ramm, G.A., Buchler, M., Friess, H., McCarroll, J.A., et al. (2004). Desmoplastic reaction in pancreatic cancer: role of pancreatic stellate cells. Pancreas *29*, 179–187.

50. Shu, S., Yang, Y., Allen, C.L., Maguire, O., Minderman, H., Sen, A., Ciesielski, M.J., Collins, K.A., Bush, P.J., Singh, P., et al. (2018). Metabolic reprogramming of stromal fibroblasts by melanoma exosome microRNA favours a pre-metastatic microenvironment. Sci. Rep. *8*, 12905.

51. Sahai, E., Astsaturov, I., Cukierman, E., DeNardo, D.G., Egeblad, M., Evans, R.M., Fearon, D., Greten, F.R., Hingorani, S.R., Hunter, T., et al. (2020). A framework for advancing our understanding of cancer-associated fibroblasts. Nat. Rev. Cancer *20*, 174–186.

52. Labernadie, A., Kato, T., Brugués, A., Serra-Picamal, X., Derzsi, S., Arwert, E., Weston, A., González-Tarragó, V., Elosegui-Artola, A., Albertazzi, L., et al. (2017). A mechanically active heterotypic E-cadherin/N-cadherin adhesion enables fibroblasts to drive cancer cell invasion. Nat. Cell Biol. *19*, 224–237.

53. Shin, H.R., and Zoncu, R. (2020). The lysosome at the intersection of cellular growth and destruction. Dev. Cell *54*, 226–238.

54. Denu, R.A., Kaur, G., Sass, M.M., Lakkaraju, A., and Burkard, M.E. (2020). Centrosome amplification in cancer disrupts autophagy and sensitizes to autophagy inhibition. Mol. Cancer Res. *18*, 33–45.

55. Peng, X., Yang, L., Ma, Y., Li, Y., and Li, H. (2020). Focus on the morphogenesis, fate and the role in tumor progression of multivesicular bodies. Cell Commun. Signal. *18*, 122.

56. Willms, E., Johansson, H.J., Mäger, I., Lee, Y., Blomberg, K.E., Sadik, M., Alaarg, A., Smith, C.I., Lehtiö, J., El Andaloussi, S., et al. (2016). Cells release subpopulations of exosomes with distinct molecular and biological properties. Sci. Rep. *6*, 22519.

57. Tauro, B.J., Greening, D.W., Mathias, R.A., Mathivanan, S., Ji, H., and Simpson, R.J. (2013). Two distinct populations of exosomes are released from LIM1863 colon carcinoma cell-derived organoids. Mol. Cell. Proteomics *12*, 587–598.

58. McKelvey, K.J., Powell, K.L., Ashton, A.W., Morris, J.M., and McCracken, S.A. (2015). Exosomes: mechanisms of uptake. J. Circ. Biomark. *4*, 7.

59. Buzás, E.I., Tóth, E.A., Sódar, B.W., and Szabó-Taylor, K.E. (2018). Molecular interactions at the surface of extracellular vesicles. Semin. Immunopathol. *40*, 453–464.

60. Willms, E., Cabañas, C., Mäger, I., Wood, M.J.A., and Vader, P. (2018). Extracellular vesicle heterogeneity: subpopulations, isolation techniques, and diverse functions in cancer progression. Front. Immunol. *9*, 738.

61. Laulagnier, K., Javalet, C., Hemming, F.J., Chivet, M., Lachenal, G., Blot, B., Chatellard, C., and Sadoul, R. (2018). Amyloid precursor protein products concentrate in a subset of exosomes specifically endocytosed by neurons. Cell. Mol. Life Sci. *75*, 757–773.

62. O'Neill, C.P., Gilligan, K.E., and Dwyer, R.M. (2019). Role of extracellular vesicles (EVs) in cell stress response and resistance to cancer therapy. Cancers (Basel) *11*, 136.

63. Eldh, M., Ekström, K., Valadi, H., Sjöstrand, M., Olsson, B., Jernås, M., and Lötvall, J. (2010). Exosomes communicate protective messages during oxidative stress; possible role of exosomal shuttle RNA. PLoS ONE *5*, e15353.

64. Li, N.F., Kocher, H.M., Salako, M.A., Obermueller, E., Sandle, J., and Balkwill, F. (2009). A novel function of colony-stimulating factor 1 receptor in hTERT immortalization of human epithelial cells. Oncogene *28*, 773–780.

65. Campeau, E., Ruhl, V.E., Rodier, F., Smith, C.L., Rahmberg, B.L., Fuss, J.O., Campisi, J., Yaswen, P., Cooper, P.K., and Kaufman, P.D. (2009). A versatile viral system for expression and depletion of proteins in mammalian cells. PLoS ONE *4*, e6529.

66. Schneider, C.A., Rasband, W.S., and Eliceiri, K.W. (2012). NIH Image to ImageJ: 25 years of image analysis. Nat. Methods *9*, 671–675.

67. Lo Cicero, A., Delevoye, C., Gilles-Marsens, F., Loew, D., Dingli, F., Guéré, C., André, N., Vié, K., van Niel, G., and Raposo, G. (2015). Exosomes released by keratinocytes modulate melanocyte pigmentation. Nat. Commun. *6*, 7506.

68. Mardakheh, F.K., Paul, A., Kümper, S., Sadok, A., Paterson, H., Mccarthy, A., Yuan, Y., and Marshall, C.J. (2015). Global analysis of mRNA, translation, and protein localization: local translation is a key regulator of cell protrusions. Dev. Cell *35*, 344–357.

69. Rappsilber, J., Mann, M., and Ishihama, Y. (2007). Protocol for micro-purification, enrichment, pre-fractionation and storage of peptides for proteomics using StageTips. Nat. Protoc. *2*, 1896–1906.

70. Tyanova, S., Temu, T., and Cox, J. (2016). The MaxQuant computational platform for mass spectrometry-based shotgun proteomics. Nat. Protoc. *11*, 2301–2319.

71. Vizcaíno, J.A., Deutsch, E.W., Wang, R., Csordas, A., Reisinger, F., Ríos, D., Dianes, J.A., Sun, Z., Farrah, T., Bandeira, N., et al. (2014). ProteomeXchange provides globally coordinated proteomics data submission and dissemination. Nat. Biotechnol. *32*, 223–226.

STAR★METHODS

KEY RESOURCES TABLE

REAGENT or RESOURCE	SOURCE	IDENTIFIER
**Antibodies**		
Rabbit Alexa-conjugated A488	Life Technologies	Cat#A11008; RRID: AB_143165
Mouse Alexa-conjugated A568	Life Technologies	Cat#A11001, RRID: AB_2534069
Rabbit Alexa-conjugated A568	Life Technologies	Cat#A11011, RRID: AB_143157
Mouse α-tubulin	Sigma-Aldrich	Cat#T9026; RRID: AB_477593
Rabbit centrin2 N-17-R	Santa Cruz	Cat#sc-27793-R; RRID: AB_2082359
Mouse LBPA (6C4)	Merck Millipore	Cat#MABT837
Rabbit LC3B (D11) XP	Cell signaling	Cat#3868S; RRID: AB_2137707
Mouse α-SMA	Sigma-Aldrich	Cat#A2547; RRID: AB_476701
Mouse Ki67 Alexa-conjugated A488	BD Biosciences	Cat#561165; RRID: AB_10611866
Rabbit TSG101 [(EPR7130(b)]	Abcam	Cat#ab125011; RRID: AB_10974262
Rabbit CD63	Abcam	Cat#ab68418; RRID: AB_10563972
Mouse CD81 (clone B-11)	Santa Cruz Biotechnology	Cat#sc-166029; RRID: AB_2275892
Mouse ALIX (clone 3A9)	Cell signaling	Cat#2171, RRID: AB_2299455
Mouse Flotillin-1 (clone 18)	Biosciences	Cat#610821; RRID: AB_398140
HRP- anti rabbit secondary	GE Healthcare	Cat#NA934; RRID: AB_772206
HRP- anti mouse secondary	GE Healthcare	Cat#NA931; RRID: AB_772210
**Chemicals, peptides, and recombinant proteins**		
Doxycycline hyclate	Sigma-Aldrich	#D9891
H2O2	Sigma-Aldrich	#H1009
N-acetyl cysteine	Sigma-Aldrich	#A9165
Bafilomycin A1	Sigma-Aldrich	#B1793-10UG
DMEM/F12	Sigma-Aldrich	#D8437
DMEM	Thermo Fisher Scientific	#41966-029
RPMI-1640	Thermo Fisher Scientific	#11875093
Keratinocyte serum free medium (1X)	Thermo Fisher Scientific	#17005042
Opti-MEM® reduced serum medium	Thermo Fisher Scientific	#31985070
DMEM for SILAC	Thermo Fisher Scientific	#88364
Penicillin/Streptomycin	Thermo Fisher Scientific	#15140-122
FBS	Thermo Fisher Scientific	#10500-064
Tet-free FBS	Hyclone	#SH30070.03T
GIBCO FBS, Dialyzed	Thermo Fisher Scientific	#26400044
Blasticidin	Generon	#2805-10
Geneticin (G418)	Thermo Fisher Scientific	#10131027
Puromycin	InvivoGen	#ant-pr-1
Polybrene	Sigma-Aldrich	#H9268
Formaldehyde 16%	Thermo Fisher Scientific	#28908
Alexa Fluor 568 Phalloidin	Life Technologies	#A12380
Hoechst 33342	ThermoFisher Scientific	# H3570
BODIPY® FL N-(2-Aminoethyl) Maleimide	Thermo Fisher Scientific	#B10250
ProLong anti-fade mounting medium	Molecular Probes	#P36934
BSA	Sigma-Aldrich	#A9647
Lipofectamine 2000	Invitrogen	#11668027
Lipofectamine RNAi Max	Invitrogen	#13778075
RIPA Buffer	Thermo Scientific	#89901
Complete Mini Protease Inhibitor Cocktail	Roche	#11836153001

(*Continued on next page*)

Continued

REAGENT or RESOURCE	SOURCE	IDENTIFIER
Phosphatase inhibitor Cocktail	Cell Signaling	#5870
Bradford Protein Assay	Bio-Rad	#5000006
Recombinant Human TGF-β1	PEPROTECH	#100-21
Matrigel ® Matrix Basement Membrane	Life Sciences	#354234
Corning Rat Tail High Concentration	Life Sciences	#354249
DTT	VWR Chemicals	#M109; CAS: 3483-12-3
Iodoacetamide	VWR Chemicals	Cat#786-228; CAS: 144-48-9
Trypsin	Sigma-Aldrich	T6567-1MG
L-Arginine	Sigma-Aldrich	A6969-25G
L-Lysine	Sigma-Aldrich	L8662-25G
L-Arginine [U-13C6]	Cambridge Isotopes	CLM-2265-H-0.5
L-Lysine [4,4,5,5-D4]	Cambridge Isotopes	DLM-2640-0.5
L-Arginine [U-13C6, U-15N4]	Cambridge Isotopes	CNLM-539-H-0.5
L-Lysine [U-13C6, U-15N2]	Cambridge Isotopes	CNLM-291-H-0.5
L-Proline	Sigma-Aldrich	P0380-100G
Critical commercial assays		
GSH/GSSH-Glo assay	Promega	#V6611
BCA Protein assay	Thermo Fisher Scientific	#23225
Magic Red Cathepsin B kit	Bio-Rad	#ICT937
CellVue Maroon Cell Labeling Kit	Invitrogen	#88-0870-16
Deposited data		
SILAC analysis of exosome secretion in response to centrosome amplification	This paper	PRIDE: PXD020984 accessible via PRIDE partner repository (https://www.ebi.ac.uk/pride/archive/)
Experimental models: cell lines		
PaTu-8988T	Prof. Hemant Kocher (QMUL)	N/A
PaTu-8988S	Prof. Yaohe Wang (QMUL)	N/A
Panc-1	Prof. Hemant Kocher (QMUL)	N/A
CFPAC-1	Prof. David Pellman (Harvard)	N/A
Capan-1	Prof. Hemant Kocher (QMUL)	N/A
HPAF-II	Prof. Hemant Kocher (QMUL)	N/A
MIA-PaCa-2	Prof. Hemant Kocher (QMUL)	N/A
DEC-hTERT	Prof. Hemant Kocher (QMUL)	N/A
HPDE	Prof. Yaohe Wang (QMUL)	N/A
PS1	Prof. Hemant Kocher (QMUL)	N/A
HEK293M	Prof. David Pellman (Harvard)	N/A
PaTu-8988S.PLK4	This work	N/A
HPAF-II.PLK4	This work	N/A
HPAF-II.PLK4 H2B RFP	This work	N/A
PS1 H2B GFP	Angus Cameron (QMUL)	N/A
Oligonucleotides		
siSAS6 on-TARGET smart pool	Dharmacon	#M-004158-02
siNegative Control	QIAGEN	#1027310
Recombinant DNA		
pLenti-CMV-TetR-Blast	Addgene	#17492
pLenti-CMV/TO-Neo-DEST.PLK4	[25]	N/A
pMD2.G VSV-G	Addgene	#12259
psPAX2 Gag-Pol	Addgene	#12260

(*Continued on next page*)

Continued

REAGENT or RESOURCE	SOURCE	IDENTIFIER
Other		
qEV original Size Exclusion Chromatography (SEC) columns	izon	#SP1
Tube, Thinwall, Ultra-Clear, 38.5 mL, 25 × 89 mm	Beckman coulter	#344058
Amicon® Ultra-15 Centrifugal Filter Unit	Merk	#UFC9010

RESOURCE AVAILABILITY

Lead contact

Further information and requests for resources and reagents should be directed to and will be fulfilled by the Lead Contact Susana Godinho (s.godinho@qmul.ac.uk).

Material availability

Plasmids and cell lines used in this work will be available upon request.

Data and code availability

All mass spectrometry raw files and search results reported in this paper have been deposited at the ProteomeXchange Consortium via the PRIDE, PRIDE: PXD020984.

EXPERIMENTAL MODEL AND SUBJECT DETAILS

Cell lines and culture conditions

Adherent cell lines were cultured at 37°C and 5% humidified CO_2. The pancreatic cancer cell lines PaTu-8988t (PaTu-T; gift from Y. Wang, BCI-QMUL) PaTu-8988s (PaTu-S), Capan-1, PANC-1, CFPAC-1, HPAF-II, MIA-PaCa-2 and DEC-hTERT (derived from normal pancreas) (gifts from H. Kocher, BCI-QMUL) were grown in DMEM supplemented with 10% FBS and 1% penicillin and streptomycin. HPDE cells (derived from normal pancreas) (gift from H. Kocher, BCI-QMUL) were grown in keratinocyte-SFM (1X) serum free media +30 μg/ml (BPE)+ 0.2ng/ml rEGF. The pancreatic stellate cell lines PS1 (gift from H. Kocher, BCI-QMUL)[64] were grown in DMEM/F12 supplemented with 10% FBS and 1% penicillin and streptomycin. 5 ng/ml of recombinant TGF-β (Peprotech) was used to treat PS1 cells for 72 hours. Tetracycline-free FBS was used to grow cells expressing the PLK4 Tet-inducible construct. STR profiling was performed for cell line authentication on the following lines: PaTu-S, PaTu-T, Capan-1, MIA-PaCa-2, Panc-1 and PS1.

METHOD DETAILS

Chemicals

Chemicals and treatments were performed as follows: 2μg/ml Doxycycline hyclate (DOX; Sigma) for 48 hours, 100 μM H_2O_2 (Sigma) for 48 hours, 5 mM N-acetyl cysteine (NAC; Sigma) for 48 hours and 20 nM Bafilomycin A1 (Sigma) for 24 hours.

Lentiviral production and Infection

To generate lentivirus, HEK293 cells were plated in antibiotic free medium. Transfection of the appropriate lentiviral plasmid in combination with Gag-Pol (psPAX2, Addgene, 12260) and VSV-G (VSV-G: pMD2.G, Addgene, 12259) was performed using lipofectamine 2000® (Thermo Fisher Scientific), as per the manufacturer's specifications. The resultant lentivirus was harvested 24 hours and 48 hours post infection, passed through a 0.4 μM syringe filter and stored in cryovials at −80°C. For infection, the appropriate lentivirus was then mixed with 8 μg/ml polybrene before being added to the cells in a dropwise fashion. Infection was repeated the following day and antibiotic selection started 24 hours after final infection.

Cells expressing the inducible PLK4 construct were generated as previously described[26]. Briefly, cells were initially infected with pLenti-CMV-TetR-Blast lentiviral vector (Addgene, 17492) and selected using Blasticidin (10 μg/ml). Post-selection, cells were then infected with a lentiviral vector containing PLK4 cDNA which had been previously cloned into the pLenti-CMV/TO-Neo-Dest vector and selected using Geneticin (200 μg/ml)[26,65]. Cells expressing the PLK4 transgene were then induced for 48 hours using 2 μg/ml of Doxycycline.

To generate H2B-RFP iPLK4 cells, lentivirus was produced by transfecting HEK293 cells with LV-RFP (Addgene 26001), pMD2.G (Addgene, 12259) and μg pCMVDR8.2 (Addgene, 12263) using FuGENE (Promega, E2311), as per manufacturer's instructions. 24 hours later the medium was replaced and 48 hours post transfection the viral supernatant was collected, passed through a 0.4 μM syringe filter and stored in cryovials at −80°C. Cells were transduced with the lentivirus as described above.

siRNA

siRNA transfection was performed in antibiotic free growth medium using Lipofectamine® RNAiMAX as per the manufacturer's specifications. For SAS-6 knock down experiments siNegative control (siNegative, QIAGEN, 1027310) and siSAS-6 (siSAS6 on-TARGET smart pool, Dharmacon, M-004158-02) were used. Per 6 well, 20 nM of siRNA was used for PaTu-S.iPLK4 cells and 50 nM for HPAF-II.iPLK4 cells as PaTu-S.iPLK4 cells were more sensitive to SAS-6 depletion and to prevent loss of centrioles below control conditions. 24 hours post transfection, the cells were trypsinized and seeded onto coverslips for analysis by immunofluorescence or into 15 cm dishes for exosome harvest experiments 72 hours post transfection.

Immunofluorescence 2D

Cells plated on glass coverslips were treated for up to 48 hours with the appropriate drug treatments, before being washed twice in PBS and fixed in 4% Formaldehyde for 20 minutes at room temperature. For centrin2 staining, cells were fixed in ice-cold methanol for 10 minutes at $-20\ ^{o}$ C. Following fixation, cells were permeabilized in 0.2% Triton X-100 in PBS for 5 minutes then blocked for 30 minutes in blocking buffer (PBS, 5% BSA, 0.1% Triton X-100). Cells were then incubated with primary antibody diluted in blocking solution for 1 hour. Cells were then washed with PBS and incubated with species-specific Alexa-conjugated secondary antibodies diluted in blocking buffer for 1 hour. Alexa Fluor 568 Phalloidin (1:250) was incubated in blocking solution for 1 hour. Cells were washed in PBS and DNA was stained with Hoechst 33342 diluted in PBS (1:5000) for 5 minutes. Finally, coverslips were mounted using ProLong Gold Antifade Mountant. Antibodies used included: Anti-centrin 2 N-17-R (Santa Cruz; 1:100), Anti α-tubulin DM1 α (Sigma-Aldrich; 1:1000), Anti LBPA 6C4 (Merck Millipore; 1:100), Anti LC3B (D11) XP ® (Cell Signaling; 1:200), Anti αSMA (Sigma-Aldrich; 1:300), Anti-Rabbit Alexa Flour 488 (Life Technologies; 1:1000), Anti-Rabbit Alexa Fluor 568 (Life Technologies 1:1000), Anti-Mouse Alexa Fluor 488 (Life Technologies 1:1000). Centrosome amplification was defined as the percentage of metaphase cells containing extra centrosomes (> 4 centrioles per cell). Images were acquired using an inverted Nikon microscope coupled with a spinning disk confocal head (Andor). Unless otherwise stated, imaging of cancer cells was performed using a 100x objective and imaging of stellate cells with a 40x objective. Images/projection images (from z stacks) were subsequently generated and analyzed with ImageJ/Fiji (National institute of Health, Bethesda, MD, USA)[66].

Immunofluorescence data analyzes

Fluorescence intensity

Where Z stack images were required to analyze fluorescence intensity, Z stack parameters were determined using the following equation: Zmin = 1.4λn/(NAobj)2. λ = the emission wavelength, n = refractive index of the immersion media, NAobj = the numerical aperture of the objective. This equation calculates the ideal z stack step size to minimize overlap between each step of the stack. Sum intensity projection images were subsequently generated using ImageJ and fluorescence intensity was quantified using ImageJ/Fiji. All conditions were quantified blindly.

LBPA-Lysotracker co-localization

To quantify co-localization, threshold images were first generated using ImageJ/Fiji. To do this, manual thresholding was performed on maximum intensity images to generate a binary image where background was removed. The images from the two channels of interest were then overlaid and the points of co-localization, white areas, were quantified manually per cell.

LBPA dispersion

LBPA dispersion was quantified using ImageJ/Fiji. The center of the nucleus for each cell was used as reference point and distance between each LBPA vesicle and nucleus was measured to assess LBPA dispersion toward the cell periphery.

LBPA size

To assess LBPA size, binary thresholding of LBPA images was first performed in ImageJ/Fiji. Threshold values were maintained in all cells/conditions. Analyze Particles function was used to calculate particle size. Maximum size was set at 700 nm to prevent quantification of LBPA aggregates.

LBPA-membrane distance

Using the z stacks for each image, orthogonal views for each cell were generated using ImageJ/Fiji. To quantify LBPA distance to the membrane, we consider only the top membrane, opposite to the coverslip. Each LBPA distance was quantified manually and classified as non-nuclear and nuclear regions (see also Figure S4I).

Extracellular Vesicle (EV) Isolation

Cells were grown for 48 hours in medium supplemented with EV depleted FBS. Vesicle depletion in FBS was performed via ultracentrifugation at 100,000 x g at 4°C for 18 hours. Where induction of centrosome amplification was necessary, cells were treated with DOX for 48 hours, before cells were washed in PBS and subsequently cultured in EV depleted media. Where drug treatments were required, cells were treated for the duration of the EV harvest (48 hours post addition of EV depleted media). After 48 hours, conditioned medium was collected, and a final cell count was performed to ensure the final cell count remained the same between cell types and conditions.

Serial ultracentrifugation (UC)

Extracellular vesicles were isolated from the conditioned media via serial ultracentrifugation steps at 4°C, similarly to[14]. Briefly, the cell culture medium was subjected to a low speed centrifugation of 500 x g for 10 minutes. The supernatant was then centrifuged at 12,000 x g for 20 minutes to pellet the large EVs ($_{L}$EVs), after removal of the supernatant the $_{L}$EVs were re-suspended in 500μl of PBS. The supernatant was then subjected to a high-speed ultracentrifugation at 100,000 x g for 70 minutes to pellet the smaller EVs ($_{S}$EVs).

The pellet was then washed in PBS and a second high-speed ultracentrifugation was performed at 100,000 x g for 70 minutes (Figure S1A). The isolated $_S$EV pellet was then re-suspended in 500 μl of PBS.

Size exclusion chromatography (SEC)

To further purify EVs isolated by serial ultracentrifugation, size exclusion chromatography (SEC) was performed using the qEV original izon science SEC columns (as per the manufacturer's instructions). Briefly, the SEC columns were equilibrated to room temperature and flushed with 5ml of buffer (PBS filtered twice through 0.22 μM filters) prior to use. 500 μl of concentrated exosomes (isolated by serial ultracentrifugation) was added to the top of the column and the eluted fractions were collected immediately in 500 μl volumes. The column was kept topped up with buffer throughout the experiment. Fractions 7-12 containing the eluted EVs were collected.

Extracellular Vesicle Quantification and Analysis

Amins ImageStream® Mark II Imaging Flow Cytometer (ImageStream)

EV samples were analyzed by ImageStream as previously described[25]. Briefly, samples were prepared in 50 μl volumes, labeled with the fluorescent lipid dye BODIPY® FL Maleimide [BODIPY® FL N-(2-Aminoethyl) Maleimide] (Thermo Fisher Scientific; 1:200) and incubated at room temperature in the dark 10 minutes prior to analysis. Samples were then loaded onto the ImageStream and vesicles were acquired at a slow flow rate with 60x magnification, a 488 nm excitation laser (BODIPY detection) and 765 nm laser (side scatter). The "remove bead" function was turned off and the flow rate allowed to stabilize before acquisitions. For acquisition, the storage gate was set to collect all events and the stopping gate set to the vesicle population (low to mid BODIPY intensity and low side scatter). The stopping gate was set to ensure that at least 20,000 objects were analyzed per acquisition. Three separate acquisitions were collected per sample. Analysis was then performed using the IDEAS software. To quantify objects/ml, a graph was generated plotting channel 02 fluorescence intensity (BODIPY) against channel 12 scatter intensity (side scatter) and a vesicle gate was re-applied to select the population at the correct BODIPY and side scatter intensities to be EVs (see Figure S1C). Where necessary the gate was adjusted using the Image library to eliminate noise and artifacts from the vesicle population. The objects/ml statistic was then used to quantify the number of objects/ml in the gated region. The average objects/ml was calculated from three separate acquisitions from each sample.

Nanoparticle tracking anlaysis

Performed using a NanoSight NS300 with a high sensitivity camera and a syringe pump. As previously described, isolated EVs were resuspended (UC) or eluted (SEC) in Dulbecco's PBS filtered twice through 0.22 μM filters. The NS300 chamber was flushed with 0.22 μM filtered deionized water and then again with 500 μl of PBS (Dulbecco's PBS filtered twice through 0.22 μM filters) to remove any particle matter. Using a 1 mL syringe 400 μl of EV sample was then flushed through the chamber until vesicles were visible on the camera to allow the focus and gain settings to be optimized. The sample was then injected into the flow cell at speed 50 and 3 recordings of 60 s each were acquired. Between samples filtered PBS was used again to flush the chamber ensuring no residual particles remained. The data was then analyzed using the NTA 3.2 analysis software and averages of the three technical replicates were plotted per experiment.

Immunolabeling electron microscopy (IEM)

A drop (5μl) of $_S$EVs (isolated by UC) suspended in PBS was deposited on formvar-carbon-coated electron microscopy grids for 20 min at room temperature, fixed with 2% paraformaldehyde in 0.2 M phosphate buffer (pH 7.4), for 20 min at room temperature, and post fixed with 1% glutaraldehyde in PBS for 5 min at room temperature. Grids containing sEV were then washed and then blocked for 5 min at room temperature in blocking buffer (PBS, 1% BSA). sEVs were then immunolabelled with a mouse anti-human CD63 primary antibody (Abcam ab23792) diluted in blocking solution for 1 hour at room temperature, washed with PBS, 0,1% BSA, incubated with a rabbit antibody against mouse Fc fragment (Dako Agilent Z0412) in PBS 0,1% BSA for 20 min at room temperature. The preparations were then immunogold labeled with protein-A gold-conjugates (10 nm; Cell Microscopy Center, Department of Cell Biology, Utrecht University). Grids were analyzed on a Tecnai Spirit G2 electron microscope (Thermo Fischer Scientific) and digital acquisitions were made with a 4k CCD camera (Quemesa, Soft Imaging System). Images were analyzed with iTEM software (iTEM CE Olympus serie) and data with Prism-GraphPad Prims software (v8)[67].

Western Blotting

Small extracellular vesicles harvested for protein extraction were isolated as previously described, however following the final ultracentrifugation, the pellet was lysed immediately in RIPA buffer supplemented with protease inhibitors on ice. To facilitate further lysis, samples were probe sonicated on ice. Protein concentration was determined using the Bio-Rad Protein Assay. 10μg of protein was loaded per well. Samples were resuspended in Laemmli buffer, resolved using the NuPAGE® Bis-Tris Electrophoresis System with NuPAGE 10% Bis-Tris Protein Gels and transferred onto PDVF membranes. Antibodies used included: Anti TSG101 EPR7130(b) (Abcam; 1:1000), Anti CD63 (Abcam; 1:1000), Anti CD81 B-11 (Santa Cruz; 1:500), Anti ALIX 3A9 (Cell Signaling; 1:1000), Anti Flotillin-1 18 (Biosciences; 1:1000), HRP- anti rabbit secondary (GE Healthcare; 1:5000) and HRP- anti mouse secondary (GE Healthcare; 1:5000). Western blots were developed on X-ray film using a SRX-101A table top film processor.

Stable isotype labeling by amino acids in cell culture (SILAC)

SILAC based proteomic analysis of exosomes was performed as previously[68]. All SILAC amino acids (heavy and medium) were purchased from Cambridge Isotopes. SILAC media and dialyzed serum were purchased from Thermo Fisher Scientific. PaTu-S.iPLK4 cells with and without the induction of centrosome amplification were grown for 6 passages in Dulbecco's modified Eagle's medium for SILAC supplemented with 10% GIBCO Dialyzed Fetal Bovine Serum (ultracentrifuged for 18 hours at 100,000 x g for EV depletion), 600 mg/L Proline and 100 mg/L of either heavy or medium Lysine and Arginine amino acids (Lys^8 and Arg^{10} for heavy, and Lys^4

and Arg[6] for medium, respectively). Labeled cells were then plated at a density of 1x10^6 cells in 40 T175 flasks per condition. 24 hours later flasks were washed in PBS and 15 mL of fresh EV depleted medium supplemented with the correct amino acids (heavy or medium) was added to the cells. 48 hours later, the conditioned medium was harvested and samples heavy and medium labeled were pooled together (Figure 2A). EVs were then isolated from the conditioned medium via ultracentrifugation and subsequent SEC as previously described. The experiment was then repeated with the labeling reversed.

Mass spectrometry

Extracellular vesicles were lysed in 8 M Urea in 50 mM Ammonium bi-carbonate (ABC) (pH 8). Samples were then sonicated using a Diagenode Bioruptor sonicator at 4°C. Samples were sonicated at high power for 15 cycles of 30 s on and 30 s off. 10 mM DTT was added for 20 minutes at room temperature followed by 55 mM Iodoacetamide incubated for 30 minutes in the dark. Protein quantification was then performed as previously described. 15 μg of protein was then selected per sample and urea was diluted to 2 M final concentration with 50 mM ABC. Samples were then subjected to in-solution trypsin digestion overnight at 25°C. The digested peptides were then acidified and desalted via stagetipping[69]. Peptides were then dried by vacuum centrifugation and resuspended in 10 μL of buffer A* (2% ACN, 0.1% trifluoroacetic acid and 0.5% acetic acid).

LC-MS/MS analysis

Equivalent of ~1 μg of each digested SILAC mix was subjected to Liquid Chromatography coupled with tandem Mass Spectrometry (LC-MS/MS), using a Q-Exactive plus Orbitrap mass spectrometer coupled with a nanoflow ultimate 3000 RSL nano HPLC platform (Thermo Fisher Scientific). Briefly, samples were resolved at a flow rate of 250 nL/min on an Easy-Spray 50 cm x 75 μm RSLC C18 column with 2 μm particle size (Thermo Fisher Scientific), using a 123 minutes gradient of 3% to 35% of buffer-B (0.1% formic acid in ACN) against buffer-A (0.1% formic acid in water), and the separated peptides were infused into the mass spectrometer by electrospray. The spray voltage was set at 1.95 kV and the capillary temperature was set to 255°C. The mass spectrometer was operated in data dependent positive mode, with 1 MS scan followed by 15 MS/MS scans (top 15 method). The scans were acquired in the mass analyzer at 375-1500 m/z range, with a resolution of 70,000 for the MS and 17,500 for the MS/MS scans. Fragmented peaks were dynamically excluded for 30 s.

Proteomics data analysis

MaxQuant (version 1.6.3.3) software was used for database search and SILAC quantifications[70]. The search was performed against a FASTA file of the *Homo sapiens*, extracted from https://www.Uniprot.org (2016). A precursor mass tolerance of 4.5 ppm, and a fragment mass tolerance of 20 ppm was applied. Methionine oxidation and N-terminal acetylation were included as variable modifications while carbamidomethylation was applied as a fixed modification. Two trypsin miss-cleavages were allowed, and the minimum peptide length was set to 7 amino acids. SILAC multiplicity was set to 3, with Lys4 and Arg6 selected as medium, and Lys8 and Arg10 as heavy labels. Minimum SILAC ratio count was set at 1. All raw files were searched together, with the match between runs option enabled. All downstream data analysis was performed by Perseus (version 1.5.5.3)[57], using the MaxQuant ProteinGroups.txt output file. Briefly, normalized SILAC H/M intensities were converted to Log 2 scale. Reverse (decoy) hits, potential contaminants, and proteins identified only by modified peptides were filtered out. Ratio values were then median subtracted. Category enrichment analysis was performed using the Fisher exact test function within Perseus. Scatterplots of the SILAC ratio values were also generated by Perseus. All mass spectrometry raw files and search results reported in this paper have been deposited at the ProteomeXchange Consortium via the PRIDE[71], with the PRIDE accession number of PXD020984.

Measuring cellular reactive oxygen species (ROS)

Cellular ROS was measured through the detection glutathione in its reduced (GSH) and oxidized (GSSG) forms using the luminescence-based GSH/GSSG-Glo Assay (Promega, V6611). Briefly, the Promega GSH/GSSG-Glo Assay is a linked assay utilizing glutathione S-transferase and Luciferin-NT that generates a luminescent signal in response to levels of GSH present in the sample. The ratio of GSH to GSSG can then be calculated to give a read out of oxidative stress in the cells, where a decrease in the ratio indicates an increase in oxidative stress. All reactions and calculations were carried out as per the manufacturer's instructions. The final ratio of GSH/GSSG was normalized to protein content to control for any changes in cell number. Protein was quantified using the Pierce BCA Protein Assay Kit (Thermo Fisher Scientific, 23227) as per the manufacturer's instructions.

Magic Red assay

The Magic Red Cathepsin B kit (Bio-Rad, ICT937) was used to analyze the protease activity of Cathepsin B in lysosomes as a proxy to lysosome function. In the presence of functional cathepsin B, the Magic Red substrate is cleaved allowing the Cresyl violet fluorophore to fluoresce red upon excitation at 550-590 nm. Briefly, cells to be analyzed were plated on coverslips and the Magic Red substrate (Magic Red stock was reconstituted in 50 μl DMSO and diluted 1:10 in deionized water) was added to the growth media (20μl was added per 300μl of growth media as per the manufacturer's instructions) for the final hour of the experiment. Cells were then fixed in 4% formaldehyde as previously described. Cresyl Violet fluorescence was detected using an inverted Nikon microscope coupled with a spinning disk confocal head (Andor). Z stack images were acquired, and sum intensity image projections were generated using ImageJ. Fluorescence intensity was then quantified per cell with ImageJ[66]. All conditions were quantified blindly.

Extracellular vesicle uptake by recipient cells

Immunofluorescence

Fluorescently labeled $_S$EVs were generated using the previously described ultracentrifugation protocol with the following alteration: prior to the final PBS wash step, $_S$EVs were resuspended in 200 μl of PBS and fluorescently labeled with BODIPY (1:200). $_S$EVs were then incubated at room temperature for 5 minutes before being diluted in 31.5 mL of PBS. The final 100,000 x g ultracentrifugation

step was then performed, and the subsequent $_S$EV pellet resuspended in 200 μl of PBS. The isolated $_S$EVs were then added to the recipient cells that had been plated on glass coverslips 24 hours prior. 3 hours post addition of $_S$EVs, coverslips were fixed in 4% formaldehyde and stained with Alexa Fluor 568 Phalloidin (1:250) and Hoechst (1:5000) as previously described. Representative z stack images were taken using a spinning disk confocal microscope as previously described.

ImageStream

$_S$EVs collected from Patu-S cells were isolated through ultracentrifugation and resuspended in 500 μl of PBS and mixed 1:1 with CellVue Maroon dye diluted in diluent C buffer (4 μl of dye is diluted in 1 mL diluent C buffer), according to the manufacturer's instructions, and incubated 5 minutes in the dark. The unbound dye was then quenched by adding 2 mL of sterile filtered PBS + 10% BSA. Samples were topped with 32 mL of PBS and ultracentrifuged for 70 minutes at 100,000 x g. $_S$EVs pellets were resuspended in 500-1000 μl of PBS. Note that we keep 1 mL of condition medium from -DOX $_S$EVs condition that we use as control to ensure that CellVue staining is the result of labeled $_S$EVs uptake. Next, $_S$EVs samples were topped to 10 mL with PBS. To remove the unbound dye, a 10 kDa molecular weight cut-off spin column (Amicon Ultra-15) was used and samples were centrifuged at 3000 x g for 10-15 minutes. Labeled $_S$EVs samples can be stored at −80C at this stage. For the uptake experiments, 0.65x10^5 PS1 cells were plated in T25 flasks 24 hours prior the addition of $_S$EVs. To ensure a similar $_S$EVs/PS1 ratio used in the PS1 activation experiments, 130 million $_S$EVs were added to each cell flask for 3 hours. Equal volume of conditioned medium collected above was used as control. After trypsinization, cells were resuspended in 50 μl of PBS and analyzed. Images of cells were acquired using an ImageStream MK-II Imaging Flow Cytometer (Millipore Sigma). Over 1000 single cells in focus per condition were analyzed using the ImageSTream Data Exploration and Analysis Software (IDEAS). Red fluorescence intensity of unstained PS1 cells were used to define gating for cells that did not internalized $_S$EVs (see Figure S5D). Cells incubated with $_S$EVs were markedly separated from the unstained cell population and showed significant increase in fluorescent intensity score as well as visible fluorescent puncta inside cells. To ensure that only cells with internalized $_S$EVs were classified as positive, a brightfield mask with 1-pixel erosion was applied. This protocol was based on previously published work[50].

Extracellular vesicle-mediated PSC activation assay

PaTu-S.iPLK4 cells untreated or induced to have amplified centrosomes (48 hours 2 μg/ml DOX treatment) were cultured for 48 hours in vesicle depleted media before the conditioned media was collected. EVs were then harvested from the conditioned media by ultracentrifugation alone, or in combination with SEC as described previously. EV number was then quantified by ImageStream as described above. 20 million EVs were then added to the culture medium of PS1 cells that had been plated on glass coverslips at a density of 1x10^4 cells 24 hours prior. 48 hours later, a second dose of 20 million EVs was administered. 24 hours later cells were fixed and stained for αSMA and DNA as described previously. Images were acquired using an inverted Nikon microscope coupled with a spinning disk confocal head (Andor) with a 40x objective. PS1 activation was quantified based on α-SMA organization, where the formation of α-SMA fibers was used as a measure of activation. Resting PS1 cells display diffuse αSMA staining consistent with low to no activation. Increase expression of αSMA with the appearance of αSMA fibers is indicative of PS1 activation and we as classify strong activation PS1 cells where the majority of αSMA is associated with fibers displaying increased intensity levels. Roughly 150 cells were quantified manually per condition. All conditions were quantified blindly.

3D co-culture spheroid invasion assay

Prior to spheroid generation, PS1 cells were either treated for 72 hours with $_S$EVs (as described above), with 5ng/ml TGF-β or left untreated. 3D spheroid cancer cell/PS1 co-cultures were generated using a hanging drop spheroid model developed by Ed Carter and Richard Grose (BCI-QMUL), based on previous work[52]. Briefly, PS1 H2B-GFP (4.4x 10^4 cells/ml) and HPAF-II.iPLK4-H2B-RFP cancer cells (2.2x10^4 cells/ml) were combined in a 0.24% methylcellulose solution (Sigma-Aldrich, M0512). Droplets containing 1000 cells were then plated on the underside of a 15 cm culture dish and left to form spheroids overnight at 37 °C. Spheroids were then collected and centrifuged at 100x g for 3 minutes before being re-suspended in gel mix solution. Gel mix solution consisted of 1.6 mg/ml Collagen I (Corning Rat Tail High Concentration) and 17.5% Matrigel ® Matrix Basement Membrane LDEV-free (Corning, 354234), prepared in PS1 culture medium and buffered to physiological pH with NaOH. Approximately 6 spheroids suspended in gel mix were added to a pre-coated well of a low attachment plate and left to solidify at 37 °C before PS1 culture medium was added on top. Spheroids were incubated for 3 days and images were taken by light microscopy. Percentage invasion was analyzed using ImageJ and calculated as a measure of the total invasive area relative to the central sphere. For confocal analyses, spheres were fixed in 4% formaldehyde prior to mounting for imaging on an LSM 880 Zeiss confocal microscope. All conditions were quantified blindly.

QUANTIFICATION AND STATISTICAL ANALYSIS

Statistical analysis

Graphs and statistics were generated using Prism 8 (GraphPad Software) where results are presented as mean ± standard deviation (SD) unless otherwise stated. Statistical analysis was performed using Student's t test, one-way ANOVA with either a Tukey's (parametric) or Kruskal-Wallis (non-parametric) post hoc test unless otherwise stated. Significance is equal to *$p < 0.05$, **$p < 0.01$, ***$p < 0.001$ and ***$p < 0.0001$.

Current Biology, Volume *31*

Supplemental Information

Centrosome amplification mediates small extracellular vesicle secretion via lysosome disruption

Sophie D. Adams, Judit Csere, Gisela D'angelo, Edward P. Carter, Maryse Romao, Teresa Arnandis, Martin Dodel, Hemant M. Kocher, Richard Grose, Graça Raposo, Faraz Mardakheh, and Susana A. Godinho

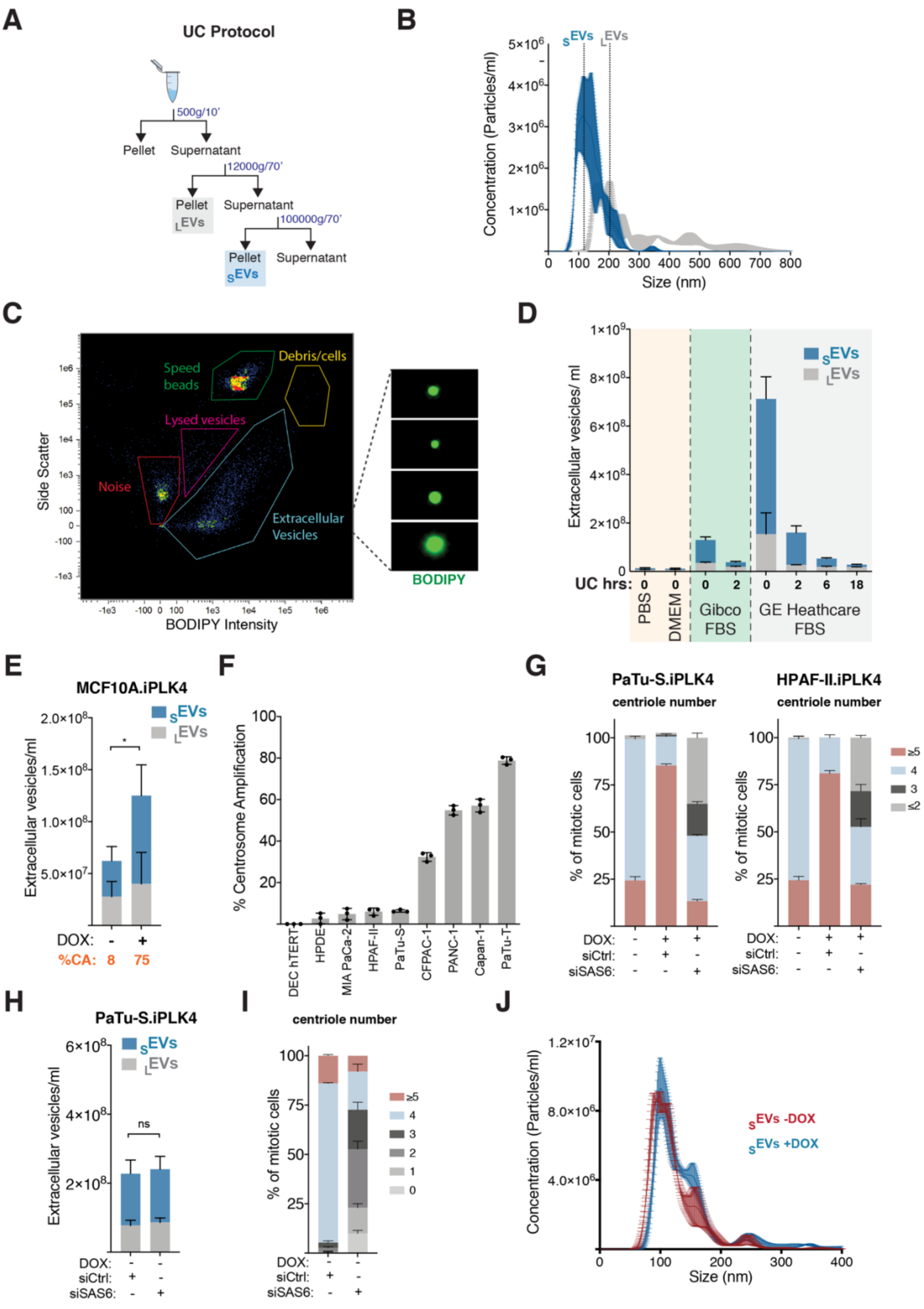

Figure S1. EV isolation and characterization in PDAC cell lines. Related to Figure 1. (A) Experimental flowchart. (B) Quantification of $_{S}$EVs and $_{L}$EVs concentration and size using the nanoparticle tracking device NanoSight to assess the reliability of the UC protocol to separate EVs by size. (C) Example scatterplot from ImageStream displaying side scatter plotted against BODIPY maleimide intensity. Representative gating regions are shown. Gating region for contaminating cells and cell debris (yellow), speed beads (green) used to internally calibrate the ImageStream and for lysed vesicles (purple) are shown. Representative images or particles taken from the ImageStream image gallery that are present in the EV gating region show spherical BOPIDY-labelled vesicles. (D) Quantification of $_{S}$EVs and $_{L}$EVs in the reagents used to culture cells and

prepare EVs for analyses and after UC to determine removal of any contaminant EV. (E) Quantification of secreted $_S$EVs and $_L$EVs from MCF10A.iPLK4 cell line upon induction of centrosome amplification (+DOX). Average of the percentage of centrosome amplification (CA) per cell line is highlighted in orange. (F) Quantification of the percentage of centrosome amplification in a panel of PDAC cell lines. n=300 mitotic cells for each cell line. (G) Quantification of the number of centrioles in the PaTu-S.iPLK4 (left) and HPAF-II.iPLK4 (right) cell lines upon induction of centrosome amplification (+DOX) and Sas-6 depletion by siRNA. n=300 mitotic cells for each condition. (H) Quantification of secreted $_S$EVs and $_L$EVs from PaTu-S.iPLK4 control cells (-DOX) upon depletion of Sas-6. 4= normal and ≥5 centrioles per mitotic cell = centrosome amplification. (I) Quantification of the number of centrioles in PaTu-S.iPLK4 cells upon depletion of Sas-6. n=300 mitotic cells for each condition. (J) Quantification of the size of $_S$EVs secreted by PaTu-S.iPLK4 cells with (+DOX) and without (-DOX) extra centrosomes using the NanoSight. For all graphics error bars represent mean +/- SD from three independent experiments. *$p < 0.05$, ****$p < 0.0001$, *n*.s. = not significant ($p > 0.05$). The following statistic were applied: for graph in E two-way ANOVA with Tukey's post hoc test was applied and for graphs in H unpaired *t* test was applied.

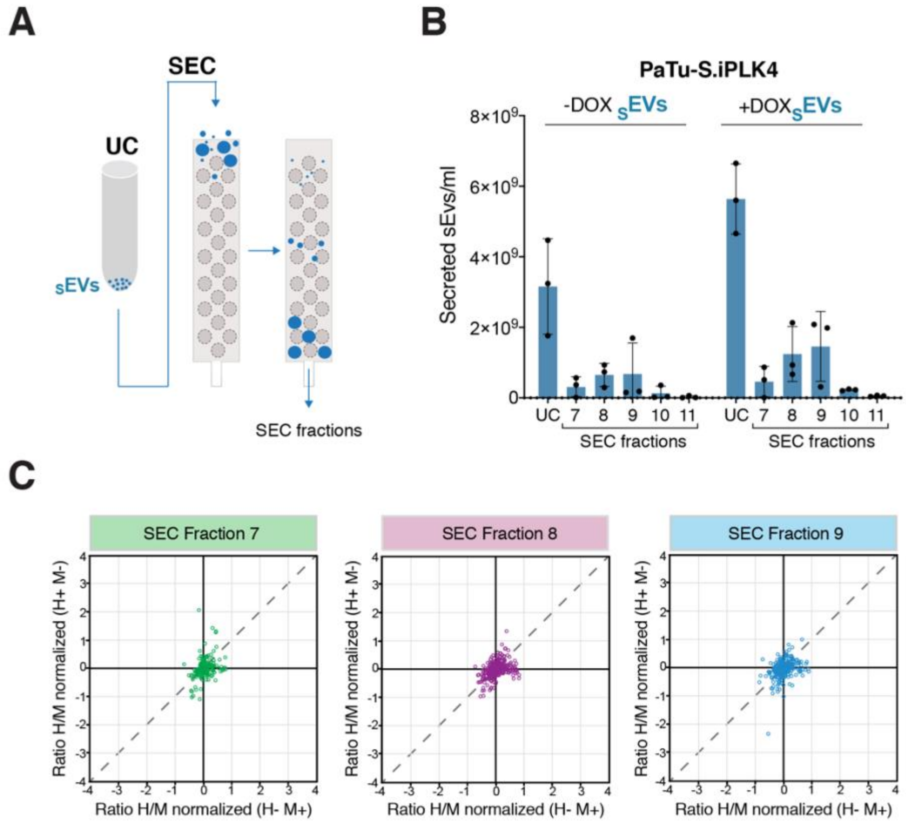

D

SEC Fraction 7			
Gene name	-DOX sEVs	+DOX sEVs	Peptide number
KIF5B	✗	✓	42
B2M	✗	✓	3
CYBRD1	✗	✓	1
ERLIN2	✗	✓	13
SLC25A3	✗	✓	14
OCLN	✗	✓	5
ANXA3	✗	✓	19
MAPK1IP1L	✓	✗	1

E

SEC Fraction 8			
Gene name	-DOX sEVs	+DOX sEVs	Peptide number
CD81	✓	✗	3
PGM3	✗	✓	8
CAD	✗	✓	48
MT-CO2	✗	✓	3
NIBAN	✗	✓	9
CCDC124	✗	✓	8

Figure S2. SILAC proteomic analyses of secreted $_S$EVs. Related to Figure 2. (A) Experimental flowchart. (B) Quantification of number $_S$EVs in UC and SEC fractions collected from PaTu-S.iPLK4 cells with (+DOX) and without (-DOX) extra centrosomes. (C) Correlation graphs plotting Log_2 fold change of the ratio of heavy (H) and medium (M) labelled proteins of the forward and reverse experiments for the SEC fractions 7, 8 and 9. Dashed diagonal line illustrates where identical M and H would lie, demonstrating the similarity between H and M labelled $_S$EVs. (D) Table with the proteins that were lost/gain in SEC fraction 7 of $_S$EVs secreted by cells with extra centrosomes (+DOX). (E) Table with the proteins that were lost/gain in SEC fraction 8 of $_S$EVs secreted by cells with extra centrosomes (+DOX). See also Table S4.

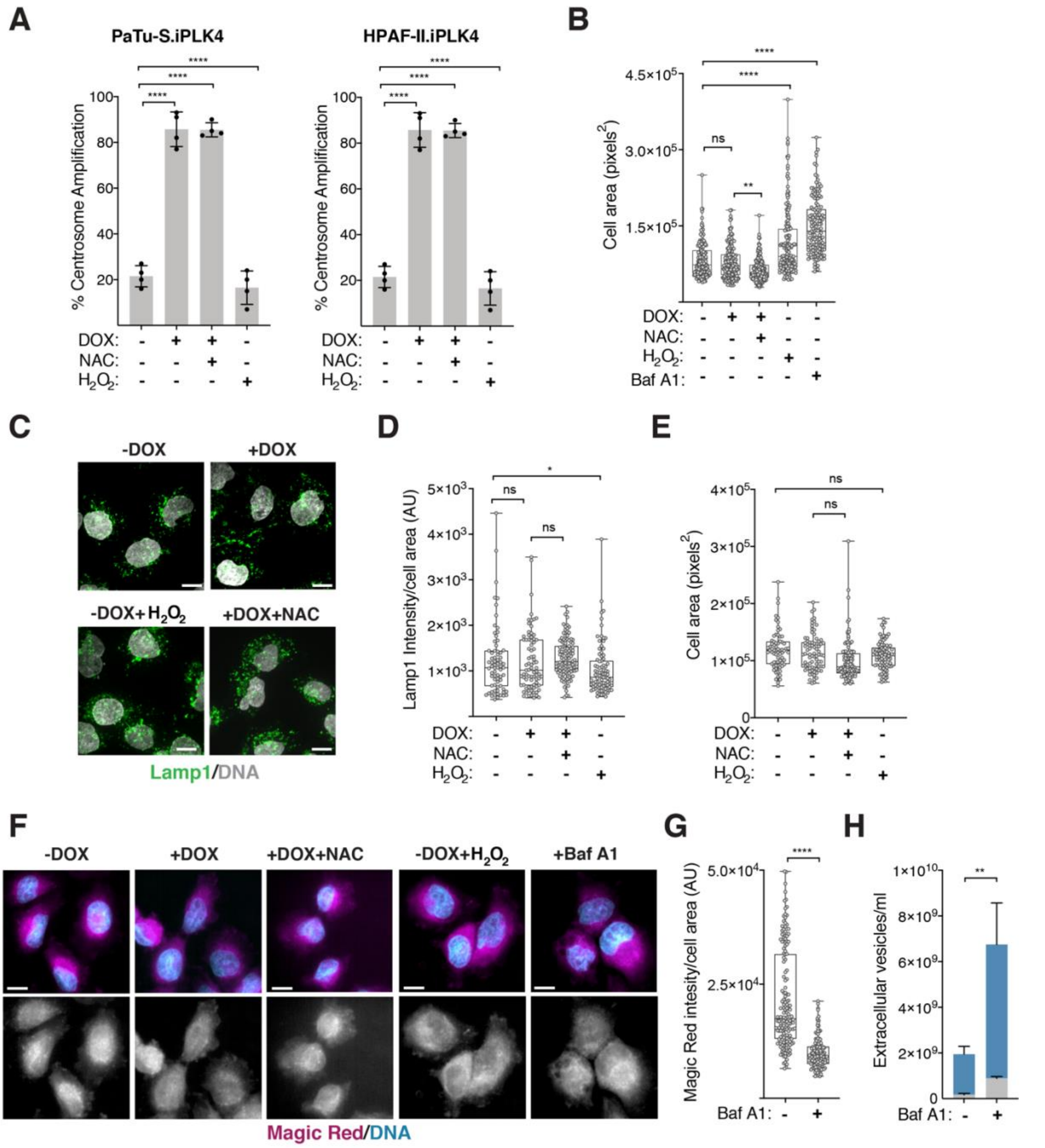

Figure S3. Characterization of lysosome function in cells with amplified centrosomes. Related to Figure 3. (A) Quantification of centrosome amplification in the PaTu-S.iPLK4 (left) and HPAF-II.iPLK4 (right) cell lines upon induction of centrosome amplification (+DOX). 5 mM of NAC, 100 µM H_2O_2 and 20 nM Baf A1 was used. n=300 mitotic cell lines for each condition. (B) Quantification of cell area (pixels^2). 5 mM of NAC, 100 µM H_2O_2 and 20 nM Baf A1 was used. $n_{(-DOX)}$=158, $n_{(+DOX)}$=189, $n_{(+DOX+NAC)}$=221, $n_{(-DOX+H2O2)}$=175 and $n_{(+BafA1)}$=144. (C) Representative confocal images of PaTu-S.iPLK4 cells stained for total lysosomes (Lamp1, green) and DNA (grey). Scale bar, 10 µm. (D) Quantification of Lamp1 fluorescence intensity in PaTu-S.iPLK4 cells normalized for cell area. 5 mM of NAC, 100 µM H_2O_2 was used. AU, arbitrary units. $n_{(-DOX)}$=71, $n_{(+DOX)}$=80, $n_{(+DOX+NAC)}$=112 and $n_{(-DOX+H2O2)}$=87. (E) Quantification of cell area (pixels^2). 5 mM of NAC, 100 µM H_2O_2 was used. $n_{(-DOX)}$=71, $n_{(+DOX)}$=80, $n_{(+DOX+NAC)}$=112 and $n_{(-DOX+H2O2)}$=87. (F) Representative confocal images of PaTu-S.iPLK4 cells stained for functional lysosomes (Magic red, magenta) and DNA (cyan). SUM projection images used for fluorescence intensity quantification. Baf A1 was used at 20 nM. Scale bar, 10 µm. (G) Quantification of intracellular Magic red fluorescence intensity normalized for cell area in PaTu-S.iPLK4 cells. AU, arbitrary units. $n_{(control)}$=158 and $n_{(+BafA1)}$=144. Note that data plotted for control cells is the same as in Figure 3D. (H) Quantification of $_{S}$EVs and $_{L}$EVs secretion in control and Baf A1 treated PaTu-

S.iPLK4 cells. For all graphics error bars represent mean +/- SD from three independent experiments. *$p < 0.05$, **$p < 0.01$, ****$p < 0.0001$, n.s. = not significant ($p > 0.05$). The following statistic were applied: for graphs in A, one-way ANOVA with Tukey's post hoc test was applied, for graphs in B, D, E and G data one-way ANOVA with a Kruskal-Wallis post hoc test was applied and for data in H two-way ANOVA with Tukey's post hoc test was applied.

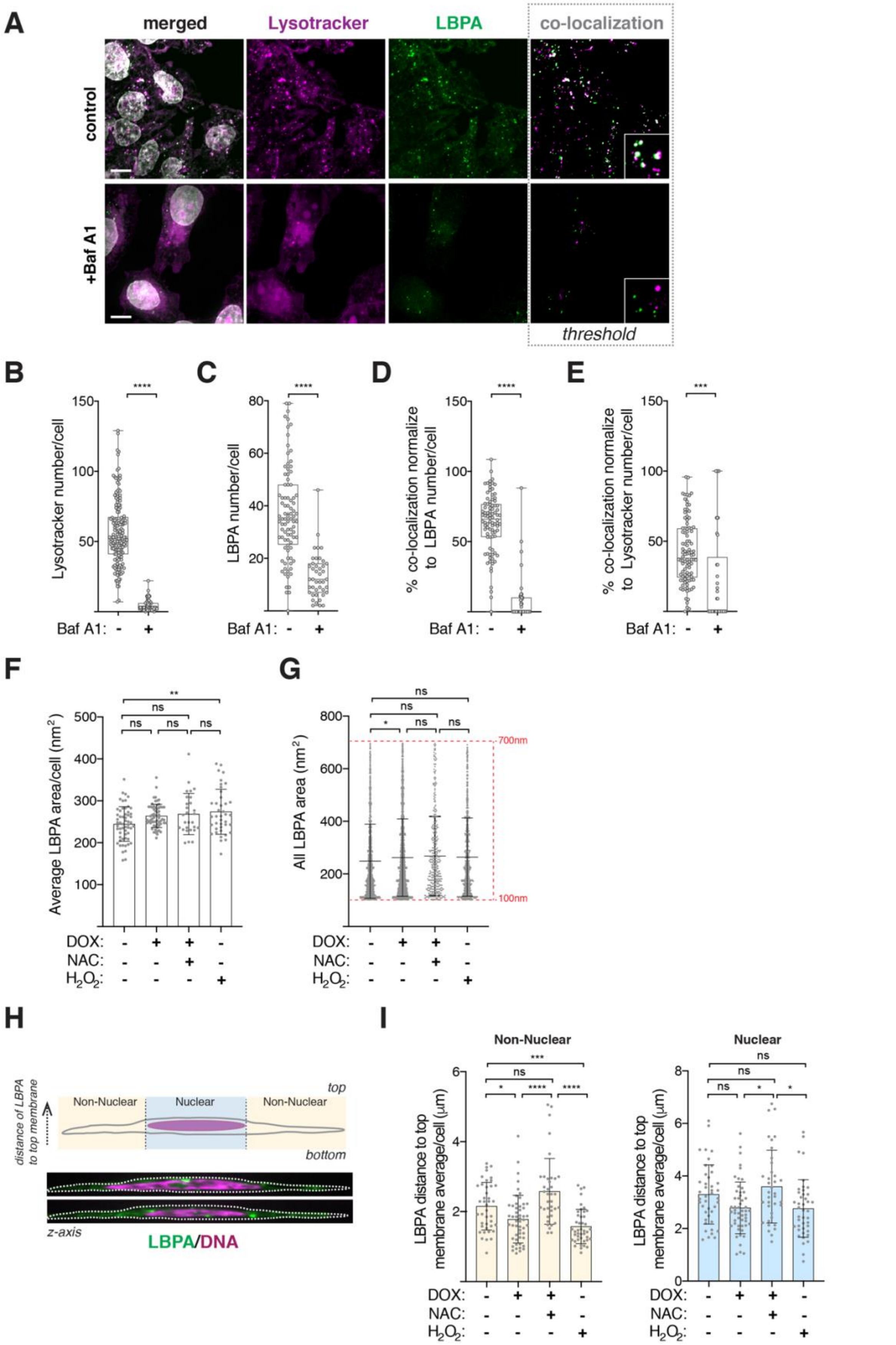

Figure S4. Characterization of Bafilomycin A1 treatment and analyses of MVB dispersion and localization. Related to Figure 4. (A) Representative confocal images of cells stained for acidic lysosomes (Lysotracker, magenta), late endosomes/MVBs (anti-LBPA, green) and DNA (grey). Insets show higher magnification of lysotracker and LBPA-labelled vesicles. Scale bar, 10 μm. (B) Quantification of the number of lysotracker-labelled lysosomes per cell. 20 nM of Baf A1 was used. $n_{(control)}$=166 and $n_{(+BafA1)}$=67. Note

that data plotted for control cells is the same as in Figure 4B. (C) Quantification of LBPA-labelled late endosomes/MVBs per cell. 20 nM of Baf A1 was used. $n_{(control)}$=88 and $n_{(+BafA1)}$=42. Note that data plotted for control cells is the same as in Figure 4C. (D) Quantification of the percentage of lysotracker and LBPA-labelled intracellular vesicles co-localization. 20 nM of Baf A1 was used. $n_{(control)}$=86 and $n_{(+BafA1)}$=42. (E) Quantification of the percentage of lysotracker and LBPA-labelled intracellular vesicles co-localization normalized to lysotracker number. 20 nM of Baf A1 was used. $n_{(control)}$=86 and $n_{(+BafA1)}$=42. (F) Quantification of the average size of LBPA vesicles per cell. $n_{(-DOX)}$=57, $n_{(+DOX)}$=63, $n_{(+DOX+NAC)}$=28 and $n_{(-DOX+H2O2)}$=40. (G) Quantification of the size of all LBPA vesicles. (H) Representative image depicting method for quantifying LPBA-membrane distance. Cells were stained for LBPA (green) and DNA (magenta). Distance is calculated to the top membrane, opposite to coverslip. (I) Left: Quantification of the average LBPA-membrane distance per cell in the non-nuclear region. Right: Quantification of the average LBPA-membrane distance per cell in the nuclear. $n_{(-DOX)}$=41, $n_{(+DOX)}$=53, $n_{(+DOX+NAC)}$=38 and $n_{(-DOX+H2O2)}$=44. Note that data plotted for control cells is the same as in Figure 4D. For all graphics error bars represent mean +/- SD from three independent experiments. *$p < 0.05$, **$p < 0.01$, ***$p < 0.001$****$p < 0.0001$, n.s. = not significant ($p > 0.05$). Graphs in B-E were analyzed with unpaired *t* test, graphs in G, and I were analyzed with one-way ANOVA with a Kruskal-Wallis post hoc test and for graph in F one-way ANOVA with Tukey's post hoc test was used.

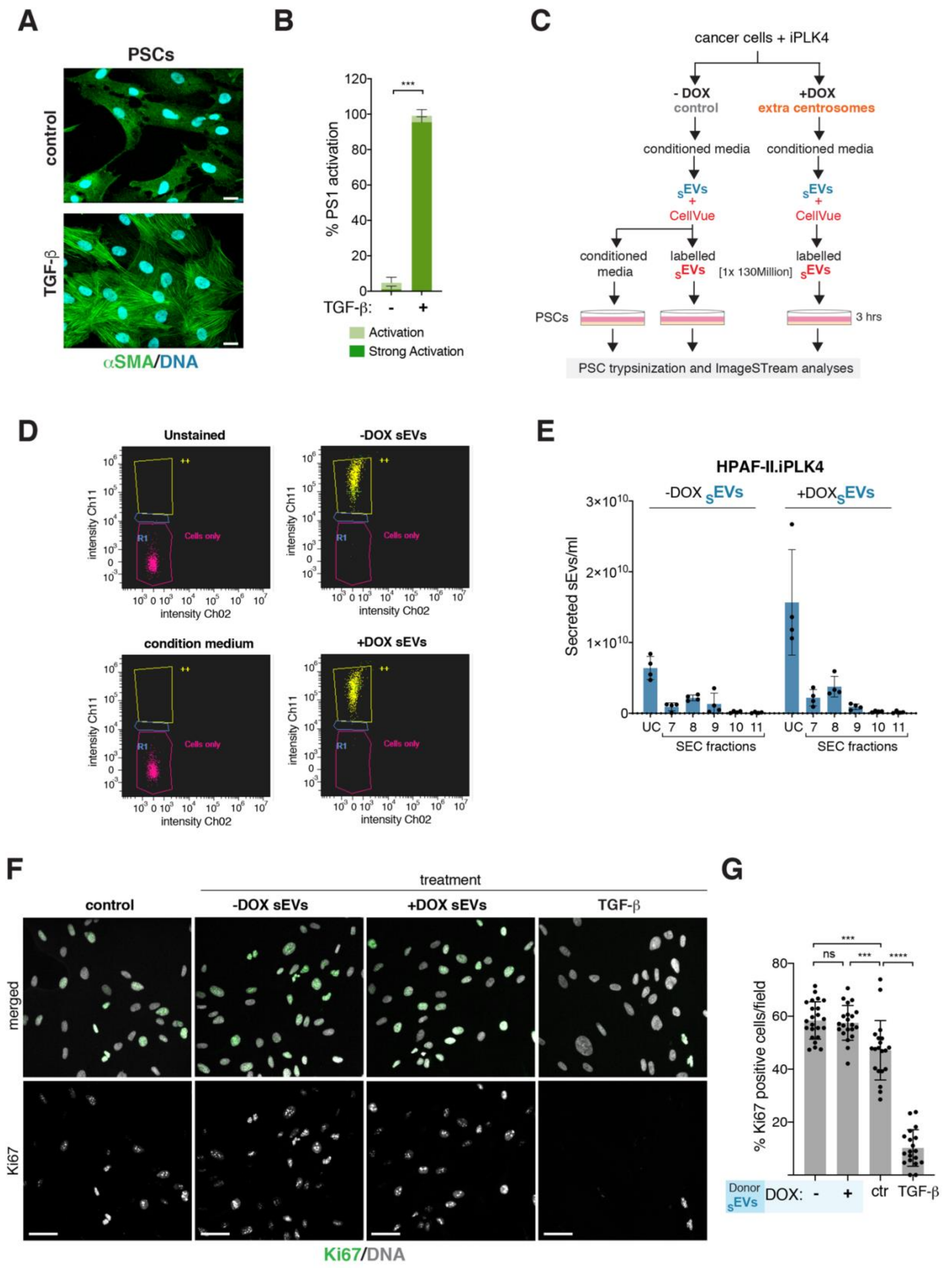

Figure S5. Characterization of PSCs activation. Related to Figure 5. (A) Representative confocal images of PSCs stained for α-SMA (green) and DNA (cyan). Scale bar, 20 μm. (B) Quantification of the percentage of PSCs activation upon treatment with TGF-β, used as positive control. 5 ng/ml of TGF-β was used. PSCs $n_{(control)}$=475, $n_{(+TGF-\beta)}$=414. (C) Schematic representation of the $_{S}$EVs uptake experiment. (D) Examples of scatterplots from ImageStream displaying unlabeled cells side scatter plotted against CellVue intensity. Representative gating regions are shown. Gating region for CellVue positive (yellow), unlabeled cells (magenta) were used to determine the percentage of cells that internalized CellVue-labelled $_{S}$EVs. (E) Quantification of number $_{S}$EVs in UC and SEC fractions collected from HPAF-II.iPLK4 cells with (+DOX) and

without (-DOX) extra centrosomes. (F) Representative confocal images of PS1 cells stained for the proliferation marker Ki67 (green) and DNA (grey). Scale bar, 50 μm. Quantification of the percentage of Ki67 positive cells per total cells in different image fields. $n_{(control)}$=842, $n_{(-DOX\ sEVs)}$=1049, $n_{(+DOX\ sEVs)}$=996 and $n_{(+TGF\text{-}\beta)}$=854. For all graphics error bars represent mean +/- SD from three independent experiments. ***$p < 0.001$. For graph in B aired *t* test was used for statistical analyses and for graph in G two-way ANOVA with Tukey's post hoc test was used.

Cell Line	Platting density in 15 cm dishes	Final cell count at end point
DEC-hTERT	3.2 $x10^6$	~6.4 $x10^6$
HPDE	3.6 $x10^6$	~6.4 $x10^6$
MIA-PaCa-2	1.32 x106	~6.4 $x10^6$
HPAF-II	1.32 $x10^6$	~6.4 $x10^6$
PaTu-S	7.5 $x10^5$	~6.4 $x10^6$
CFPAC-1	1.44 $x10^6$	~6.4 $x10^6$
Panc-1	2.16 $x10^6$	~6.4 $x10^6$
Capan-1	2.52 $x10^6$	~6.4 $x10^6$
PaTu-T	1.2 x106	~6.4 $x10^6$
PaTu-S.iPLK4 -DOX	7.5 $x10^5$	~6.4 $x10^6$
PaTu-S.iPLK4 + 48 hrs DOX	8 $x10^5$	~6.4 $x10^6$
HPAF-II.iPLK4 -DOX	1.32 $x10^6$	~6.4 $x10^6$
HPAF-II.iPLK4 + 48 hrs DOX	1.35 $x10^6$	~6.4 $x10^6$

Table S1. Cell platting conditions. Related to Figure 1. Platting density in 15 cm dishes utilized for the different cell lines and conditions to ensure a similar final cell number at end point, when EVs are collected for analyses. Note that for all experiments, final cell numbers were always assessed.
